# Endoplasmic Reticulum Stress and the Unfolded Protein Response in Cerebral Ischemia/Reperfusion Injury

**DOI:** 10.3389/fncel.2022.864426

**Published:** 2022-05-04

**Authors:** Lei Wang, Yan Liu, Xu Zhang, Yingze Ye, Xiaoxing Xiong, Shudi Zhang, Lijuan Gu, Zhihong Jian, Hongfa Wang

**Affiliations:** ^1^Department of Neurosurgery, Renmin Hospital of Wuhan University, Wuhan, China; ^2^Department of Anesthesiology, Renmin Hospital of Wuhan University, Wuhan, China; ^3^Central Laboratory, Renmin Hospital of Wuhan University, Wuhan, China; ^4^Rehabilitation Medicine Center, Department of Anesthesiology, Zhejiang Provincial People’s Hospital, Affiliated People’s Hospital, Hangzhou Medical College, Hangzhou, China

**Keywords:** ER stress, unfolded protein response (UPR), cerebral ischemia-reperfusion injury (CIRI), inflammation, apoptosis

## Abstract

Ischemic stroke is an acute cerebrovascular disease characterized by sudden interruption of blood flow in a certain part of the brain, leading to serious disability and death. At present, treatment methods for ischemic stroke are limited to thrombolysis or thrombus removal, but the treatment window is very narrow. However, recovery of cerebral blood circulation further causes cerebral ischemia/reperfusion injury (CIRI). The endoplasmic reticulum (ER) plays an important role in protein secretion, membrane protein folding, transportation, and maintenance of intracellular calcium homeostasis. Endoplasmic reticulum stress (ERS) plays a crucial role in cerebral ischemia pathophysiology. Mild ERS helps improve cell tolerance and restore cell homeostasis; however, excessive or long-term ERS causes apoptotic pathway activation. Specifically, the protein kinase R-like endoplasmic reticulum kinase (PERK), activating transcription factor 6 (ATF6), and inositol-requiring enzyme 1 (IRE1) pathways are significantly activated following initiation of the unfolded protein response (UPR). CIRI-induced apoptosis leads to nerve cell death, which ultimately aggravates neurological deficits in patients. Therefore, it is necessary and important to comprehensively explore the mechanism of ERS in CIRI to identify methods for preserving brain cells and neuronal function after ischemia.

## Introduction

Ischemic stroke, which accounts for approximately 87% of all stroke cases (Kuriakose and Xiao, 2020), results in severe symptoms and is responsible for the majority of stroke-related deaths and disabilities. The main cause of ischemic stroke is cerebrovascular blockade, which leads to brain dysfunction in the corresponding region. As the disability rate and mortality rate of ischemic stroke are very high, this disease seriously affects the health of individuals and imposes a large burden on society and the economy (Poustchi et al., 2021). The amount of glucose and glycogen stored in brain tissue is very low, making the brain very sensitive to reduced blood flow, which can lead to irreversible damage after 20 min (Kristian, 2004). Compared with other organs, the brain is rich in polyunsaturated fatty acids (FAs) but contains very low levels of protective antioxidants such as superoxide dismutase and catalase. Thus, it is very sensitive to oxidative stress injury (Adibhatla and Hatcher, 2010). During ischemic stroke, cerebral blood flow is interrupted or reduced, resulting in hypoxic and ischemic damage to brain cells, cell necrosis, or cell apoptosis. During ischemia, anaerobic metabolism dominates in tissues, and adenosine triphosphate (ATP) levels decrease rapidly. Lactate accumulates, leading to a decrease in the intracellular pH value, leading to an imbalance in ATP-dependent ion transport, overload of intracellular calcium ions, and swelling and rupture of cells, ultimately mediating cell death through necrosis, apoptosis, and autophagy (Kalogeris et al., 2012).

At present, the methods for achieving vascular recanalization in patients with ischemic stroke mainly include the use of recombinant tissue plasminogen activator (rtPA) and vascular interventional thrombectomy. Basic and clinical research has led to improvements in the treatment of ischemic stroke. Intravenous rtPA is the recommended treatment for acute cerebral infarction within 4.5 h of onset (Man et al., 2020; Powers, 2020). However, due to time constraints, the existing treatment methods are limited. Importantly, ischemic stroke may lead to intracranial hemorrhage (ICH), cause additional brain injury, and even endanger the patient’s life. When blood flow is restored to the brain after a certain period of time, brain injury and brain dysfunction are often aggravated. This phenomenon, called cerebral ischemia/reperfusion injury (CIRI) (Sun et al., 2018), occurs because although oxygen levels are restored to normal after reperfusion, reactive oxygen species (ROS) are produced during this process, and infiltration of proinflammatory neutrophils into ischemic tissues aggravates ischemic injury, eventually leading to mitochondrial permeability transition (MPT) pore opening and further irreversible damage (Kalogeris et al., 2012). The pathophysiological process of CIRI is complex and involves a variety of different mechanisms, including oxidative stress, inflammation, intracellular Ca^2+^ overload, mitochondrial dysfunction, apoptotic cell death, and excitatory amino acid toxicity (Kalogeris et al., 2016; Campbell et al., 2019; Datta et al., 2020). These factors are interrelated and interact with each other to eventually cause nerve cell death and neurological dysfunction. Recent studies have shown that CIRI can also cause endoplasmic reticulum (ER) damage and dysfunction, activate downstream signaling pathways, contribute to ischemia/reperfusion injury, and have an important impact on nerve cell apoptosis and survival (Hetz and Saxena, 2017).

The ER is an organelle that is found in all eukaryotic cells except mature red blood cells and is mainly responsible for the secretion and folding of proteins, the storage and release of calcium, the synthesis and distribution of lipids, and other functions (Stefan et al., 2011; Oakes and Papa, 2015; Addinsall et al., 2018). However, the ER is also sensitive to the environment. In the presence of abnormal energy metabolism, changes of glycosylation, disorder of calcium balance, drugs, toxins, and other influencing factors, the function of the ER will be impaired, leading to the aggregation of misfolded proteins and endoplasmic reticulum stress (ERS) (Guan et al., 2014). Moreover, the ER is one of the earliest organelles in cells to respond to external stress. There are three responses associated with ERS, namely, the unfolded protein response (UPR), the endoplasmic reticulum overload response (EOR), and the sterol regulatory element-binding protein (SREBP) pathway regulation response (Pahl, 1999). ERS most commonly involves the UPR, which helps cells adapt to changes in the intracellular microenvironment by altering the functional state of the ER (Markouli et al., 2020). When ERS is caused by changes in the internal and external environment, the UPR is initiated to alleviate the harmful effects caused by ERS and maintain intracellular homeostasis. The UPR involves a reduction in translational activity, an increase in protein folding ability, and activation of the protein degradation pathway. Particularly, the ER-associated degradation (ERAD) or the ubiquitin–proteasome system (UPS) (Sanderson et al., 2015; Sprenkle et al., 2017). The function of the UPR depends on the stress level. When the degree of ERS is low or the duration is short, the purpose of the UPR is to restore ER homeostasis, but when the degree of ERS is high or the duration is long, the main purpose of the terminal stage of the UPR is promotion of apoptosis (Walter and Ron, 2011; Hetz and Papa, 2018). The UPR regulates the transcription and translation of proteins in cells to alleviate harm and reduce the probability of protein misfolding. If this mechanism cannot achieve its purpose, inflammatory and apoptotic pathways may be activated, leading to the exacerbation of the inflammatory response in the nervous system, affecting cell survival (Bellezza et al., 2014; Logsdon et al., 2016).

Endoplasmic reticulum stress plays a key role in the progression of CIRI (Xin et al., 2014; Yang and Paschen, 2016). Severe CIRI disrupts ER homeostasis and leads to cell death (Luchetti et al., 2017). The function of early ERS is to restore the stability of the internal environment of the ER and protect cells. Transient and mild ERS helps cells reestablish homeostasis. However, long-term severe ERS disrupts cell homeostasis, leading to apoptosis and aggravating brain injury (Szegezdi et al., 2006; Chi et al., 2019). ERS signals are transmitted through three UPR receptors, i.e., inositol-requiring enzyme 1 (IRE1), protein kinase R-like endoplasmic reticulum kinase (PERK), and activating transcription factor 6 (ATF6), to enter the ER. These receptors bind glucose-regulated protein 78 (GRP78)/Bip (also known as HSP5A) on the ER membrane, which maintains them in an inactive state. Under unstressed conditions, GRP78/Bip binds ATF6, IRE1, and PERK to prevent them from activating downstream signaling events. When the amount of unfolded or misfolded proteins increases, Bip dissociates from these receptors and helps fold unfolded or misfolded proteins, resulting in activation of these receptors and downstream signaling events (Zhang and Kaufman, 2006). Cerebral ischemia causes a series of pathophysiological processes in which ERS-mediated apoptosis eventually leads to brain cell death (Zhao et al., 2018). Therefore, strategies that can effectively regulate ERS may be useful for the treatment of cerebral ischemia. Elucidating the interaction between the ER, cerebral ischemia, and the underlying mechanism is important for the development of effective treatments for cerebral ischemia.

## Factors Related to Cerebral Ischemia/Reperfusion Injury-Induced Endoplasmic Reticulum Stress

### Ca^2+^ Overload

Ca^2+^ plays an important role in a variety of pathophysiological processes in cells, such as gene expression, protein synthesis and transport, and cell proliferation and differentiation (Clapham, 2007). The ER and mitochondria interact and influence each other and can form physical contact points called mitochondria-associated endoplasmic reticulum membranes (MAMs) (Hayashi et al., 2009). The ER also contacts the plasma membrane (PM), and the interaction between the ER and PM is controlled by Ca^2+^ levels (Toulmay and Prinz, 2011). In the ER, calcium is needed to activate calcium-dependent molecular chaperones that can stabilize protein folding intermediates (Kim et al., 2008). Thus, it can affect ERS.

Ca^2+^ homeostasis disruption in the ER plays a decisive role in many neurological diseases, including stroke (Paschen and Mengesdorf, 2005). During cerebral ischemia, many mechanisms can cause an increase in the intracellular Ca^2+^ content. During cerebral ischemia occurs, the brain mainly relies on glucose-independent degradation to generate ATP due to the lack of oxygen and energy in nerve cells, leading to the aggregation of lactate, hydrogen ions, and nicotinamide adenine dinucleotide and a decrease in the intracellular pH. To restore a normal pH, H^+^ is excreted via Na^+^/H^+^ exchange, which in turn leads to Na^+^ inflow. However, the increase in Na^+^ content is prevented by the Na^+^/Ca^2+^ exchanger, which increases intracellular Ca^2+^ levels. During hypoxia, the sarco/endoplasmic reticulum Ca^2+^-ATPase (SERCA) is impaired, reducing the uptake of calcium by the ER and increasing the release of calcium. This further aggravates intracellular calcium overload and seriously affects the calcium storage function of the ER, leading to disruption of ER homeostasis (Sanada et al., 2011). Furthermore, due to the large increase in ROS levels, intracellular Ca^2+^ content is markedly increased during reperfusion (Baines, 2009). In addition, nitric oxide (NO), which promotes the release of calcium ions from the ER into the cytoplasm that eventually leads to calcium overload, is produced during ischemia and hypoxia (Rajakumar et al., 2016).

When the concentration of Ca^2+^ reaches a lethal level in cells, a series of changes are triggered, and damage is aggravated (Kalogeris et al., 2012). First, some Ca^2+^ is transported into the mitochondria through unidirectional transport, but once the concentration of Ca^2+^ in mitochondria exceeds the tolerated level, MPT pore opening occurs. Second, a pathologically high concentration of Ca^2+^ in the cytoplasm leads to activation of Ca^2+^/calmodulin-dependent protein kinases (CaMKs), which aggravates cell death and organelle dysfunction. Third, a high concentration of Ca^2+^ can increase the activity of calpain, promote protein translation, and lead to cell death. Fourth, a high concentration of calcium in cells can lead to the production of calcium pyrophosphate complexes and uric acid, which can combine with protein complexes in cells to form inflammasomes to promote the production of inflammatory factors and ultimately alter the inflammatory response. A high calcium concentration in the cytoplasm and a low calcium concentration in the ER and extracellular environment causes inactivation of a variety of calcium-dependent proteases, resulting in ERS.

### Free Radicals

Free radicals include ROS and reactive nitrogen species (RNS). Normally, ROS and RNS play regulatory roles in ERS. The sources of ROS in different human tissues are different. The main sources of ROS in the brain are NADPH oxidase (NOX), mitochondria, xanthine oxidase (XO), and monoamine oxidase (MAO) (Granger and Kvietys, 2015). In the reperfusion phase of cerebral ischemia, the enzyme NOX uses oxygen as the final electron receptor through NADPH, leading to immediate production of O^2–^ which is involved in the degradation of NO and protein tyrosine nitration (Wu M. Y. et al., 2018). Mitochondria are also a main source of ROS in addition to generating energy and regulating cell signals and apoptosis (Murphy, 2009). MAO is located in the outer mitochondrial membrane and helps increase H2O2 production (Granger and Kvietys, 2015).

During ischemia/reperfusion injury, excessive ROS may lead to cell death through autophagy, necrosis, and apoptosis (Cursio et al., 2015). ROS can effectively trigger ERS, and severe ERS can lead to apoptosis during CIRI (Shi et al., 2019; Wei et al., 2019). Excessive ROS act on the ER, leading to depletion of calcium ions in the ER and entry of calcium ions into cells, which eventually causes calcium overload in cells, thereby aggravating ERS and inducing apoptosis. Some studies have shown that the ER and ROS interact through some factors and signaling pathways, including glutathione (GSH)/glutathione disulfide, NOX4, and Ca^2+^ (Cao and Kaufman, 2014).

After cerebral ischemia/reperfusion, oxygenated blood reenters the ischemic tissue and cause the production of a large amount of ROS. ROS can modify almost all biomolecules in cells, which leads to cell dysfunction (Raedschelders et al., 2012). At present, ROS mainly cause damage in the following three ways. First, they oxidize or nitrify key proteins involved in regulating cell signaling through the formation of covalent bonds (Lima et al., 2010). Second, reactive nitrogen/oxide species (RNOS) directly cause cell damage. Third, oxidants, such as hydrogen peroxide, cause indirect damage via regulation of signals in dysfunctional cells and regulation of the sulfhydryl redox cycle (Go et al., 2010). During ischemia, NO is produced via oxidation of arginine to citrulline, nitrite, nitrite reduction, and mitochondrial cytochrome c (Cyt c) oxidase under hypoxic conditions (Golwala et al., 2009). During the reperfusion stage, the amount of NO produced by ischemic tissue increases, and nitrite peroxide is produced in the ER. Nitrite peroxide is highly toxic and may affect the function of some proteins.

### Inflammation

Endoplasmic reticulum stress and non-infectious inflammatory reactions are involved in many diseases. The inflammatory response participates in the pathophysiological process of CIRI, leading to cell death (Su et al., 2017). In contrast, some researchers have found that inhibiting the inflammatory response can reduce the infarct volume, improve neurological function scores, and protect brain function in rats with middle cerebral artery occlusion (MCAO) (Liu et al., 2018c). Furthermore, a recent study on CIRI showed that local inflammation is one of the main causes of ERS. After cerebral ischemia/reperfusion, microglia release interleukin (IL)-1β, IL-6, and tumor necrosis factor-α (TNF-α). These proinflammatory cytokines promote the aggregation of inflammatory cells and the production of more inflammatory cytokines, which further aggravate brain function impairment (Mo et al., 2020).

Changes in cell permeability, cell edema, inflammation, and ERS are the main processes in early cerebral ischemia. After reperfusion, blood circulation is restored, and oxygen and neutrophils reach the ischemic tissue. However, some tissues are necrotic, leading to aggravation of neutral cell aggregation and the production of ROS-dependent mediators. These mediators can promote leukocyte adhesion to the posterior vein of the capillary wall and enter the tissue to aggravate injury (Kvietys and Granger, 2012). Activation of TNF, IL-6, and IL-8 further induces ERS (Lee et al., 2019). Nuclear factor κB (NF-κB) plays a key role in the immune response and can promote the expression of inflammatory factors. In addition, ERS can promote the activation of the NF-κB signaling pathway and promote inflammation (Adolph et al., 2013). In response to ERS, eukaryotic initiation factor 2α (eIF2α) phosphorylation reduces global mRNA translation and stimulates NF-κB transcription. Inhibition of mRNA translation can reduce the protein levels of inhibitor of nuclear factor κB (IκB) and NF-κB (Deng et al., 2004). Studies have shown that eIF2α phosphorylation can inhibit the expression of IκB and activate the NF-κB pathway. Some scholars speculate that this may be because the half-life ratio of IκB to NF-κB is short, causing an increase in the proportion of NF-κB relative to IκB, leading to NF-κB nuclear translocation (Sprenkle et al., 2017). NF-κB is an inflammation-related cytokine that promotes the inflammatory response, leading to the overexpression of iNOS, IL-1β, and IL-6, aggravating CIRI (Sun et al., 2014). In turn, ERS can also be regulated by the NF-κB signaling pathway. Sphingosine kinase 1 (SPHK1) is a novel regulator of ERS. One study showed that SPHK1 can activate the NF-κB pathway, causing ERS (Zhang et al., 2020).

Endoplasmic reticulum stress can also affect inflammation. Recent studies have reported that ERS can regulate TNF-α, IL-12, and matrix metalloproteinase-12 expression. In addition, a study showed that the inositol-requiring enzyme 1α (IRE1α)-X box-binding protein 1 (XBP1) pathway can activate NLRP3 inflammasome-mediated inflammation. Particularly, XBP1 can activate the NLRP3 inflammasome, convert inactive caspase-1 into active caspase-1, and promote the conversion of IL-1β precursor into the active form of IL-1β, causing its secretion into the extracellular space (Yue et al., 2016). ROS produced by mitochondria can consistently activate the NLRP3 inflammasome and affect the function of mitochondria. Inhibition of NLRP3 activation can reduce neuronal injury and exert a neuroprotective effect after CIRI (Guo et al., 2018), while ERS and autophagy promote the death of neurons after cerebral ischemia through the NLRP3 inflammasome (Xu et al., 2021).

Therefore, ERS and the inflammatory response have a causal relationship. However, the mechanism underlying the interaction between ERS and inflammation in specific environments is still unclear. In addition, the crosstalk between ERS and inflammation in neurons, astrocytes, and microglia continues to be elucidated (Sprenkle et al., 2017). It is worth noting that the role of inflammation in CIRI has received increasing attention.

## Signal Transduction Pathways Involved in Endoplasmic Reticulum Stress

**FIGURE 1:**
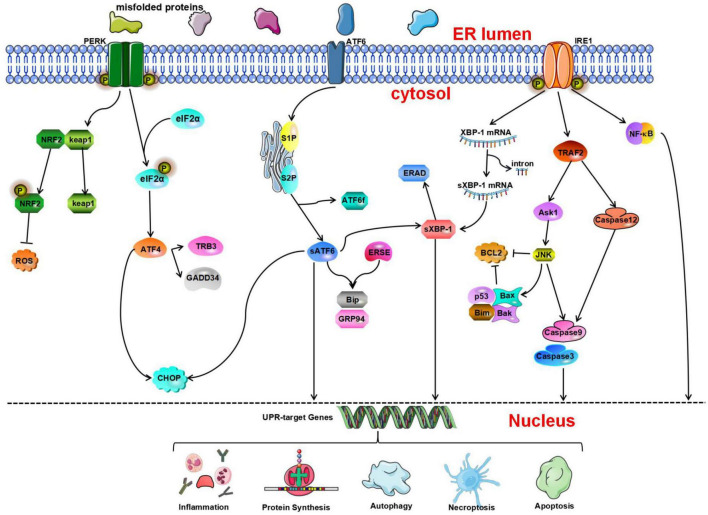
The unfolded protein response (UPR) determines cell fate through the protein kinase R-like endoplasmic reticulum kinase (PERK), inositol-requiring enzyme 1 (IRE1), and activating transcription factor 6 (ATF 6) pathways. Nuclear factor erythroid 2-related factor 2 (NRF2) is phosphorylated by PERK and dissociates from Kelch-like ECH-associated protein 1 (Keap1) under oxidative stress conditions and then activates the expression of NRF2-dependent antioxidant genes. p-eIF2a can inhibit protein synthesis. Activated ATF4 induces the expression of growth arrest and DNA damage-inducible gene 34 (GADD34) and tribbles-related protein 3 (TRB3). ATF6 is cleaved by serene protease site 1 protease and site 2 protease (S1P and S2P, respectively) to generate ATF6f and activated sATF6. Then, it combines with endoplasmic reticulum stress response elements (ERSEs) to regulate and activate the expression of BiP and glucose regulating protein 94 (GRP94). In addition, IRE1 contributes to ERS-mediated apoptosis through the tumor necrosis factor receptor-associated factor 2- activate apoptosis signal-regulating kinase-1-c-Jun N-terminal kinase (TRAF2-ASK1-JNK) and caspase-12 pathways. In addition, inositol-requiring enzyme 1α (IRE1α) can activate the nuclear factor κB (NF-κB) signaling pathway to initiate inflammatory reactions.

Blockage of cerebral blood flow causes the initiation of the UPR followed by impairment of ER or cell function. The UPR involves many enzymes and transcription factors. To date, three ER transmembrane receptors, i.e., PERK, IRE1, and ATF6, which mediate three different signaling pathways that affect transcription and translation, have been identified (Schonthal, 2012; Gupta et al., 2016; Almanza et al., 2019). Under physiological conditions, these three proteins bind to the ER chaperone GRP78. The physical binding of GRP78 to these ER transmembrane proteins maintains the proteins in an inactive state. Under physiological conditions, these three transmembrane receptors bind to the ER molecular chaperone GRP78/Bip, inhibiting their functions (Bertolotti et al., 2000). During ERS, GRP78 dissociates from these transmembrane receptors and binds aggregated unfolded proteins. Then, PERK, IRE1, and ATF6 are autophosphorylated, and their signaling pathways are activated, leading to initiation of the UPR and maintenance of ER function (Figure 1; Volmer et al., 2013; Ibrahim et al., 2019). The activation of the three branches of the UPR leads to the formation of a complex signaling network that contributes to cellular processes such as protein folding, protein degradation, and cellular redox reactions. Misfolded proteins are degraded in the cellular matrix through a process called ERAD (Lopata et al., 2020). Ubiquitination of a substrate can promote its rapid hydrolysis. This helps to maintain the dynamic balance of the ER. In general, activated IRE1 and cleaved ATF6 are involved in XBP1-induced ERAD (Waldherr et al., 2019). The PERK, IRE1, and ATF6 signaling pathways are protective pathways as they relieve early ERS. When harmful stimuli or long-term stimulation impairs ER function, the ERS-mediated cell death pathway, autophagy, apoptosis, and related inflammatory reactions can be induced.

### The PERK Pathway

Protein kinase R-like endoplasmic reticulum kinase is a type I transmembrane protein kinase located on the ER membrane (Harding et al., 1999). Its C-terminus contains a serine/threonine protein kinase domain found in upstream members of the eIF2α kinase family. During ERS, because unfolded or misfolded proteins in the ER competitively bind GRP78, GRP78 dissociates from PERK, resulting in disinhibition of PERK and activation of PERK through dimerization and autophosphorylation (McQuiston and Diehl, 2017). Activated PERK phosphorylates eIF2α, inhibits protein translation, and reduces the aggregation of unfolded proteins in the lumen of the ER (Harding et al., 2000). Phosphorylation of eIF2α can prevent the translation of mRNA (Starck et al., 2016). In the ER-related apoptotic pathway, phosphorylated PERK and eIF2α are significantly activated. It has been confirmed that during early ischemia/reperfusion, phosphorylation of eIF2α by PERK, which is the main mechanism through which the translation of proteins is inhibited during stresses, increases markedly (Owen et al., 2005; Gu et al., 2020). In addition to inhibiting protein translation, phosphorylated eIF2α can also activate the expression of activating transcription factor 4 (ATF4) (Harding et al., 2000). ATF4 is a member of the leucine zipper family and activates the basic region of transcription factors. It is a stress response gene and participates in the UPR. Under normal conditions, the content of ATF4 is very low, and ATF4 mRNA is rarely translated. In addition, some studies have shown that the transcription of ATF4 is dependent on phosphorylated eIF2α (Blais et al., 2004). ATF4 can activate two survival and apoptosis pathways during the UPR. ATF4 binds its activator to form a complex, which combines with the promoter of the survival-promoting gene GRP78 (Mamady and Storey, 2008). In addition, activated ATF4 induces the expression of CAAT/enhancer-binding protein (C/EBP) homologous protein (CHOP) (Palam et al., 2011; Han et al., 2013), growth arrest, DNA damage-inducible gene 34 (GADD34) (Ma and Hendershot, 2003), and tribbles-related protein 3 (TRB3) (Ohoka et al., 2005), which promote the initiation of apoptosis. The p-eIF2α-induced decrease in translation reduces the protein load in the lumen of the ER, while adaptive gene expression induced by ATF4 involves amino acid metabolism and protein homeostasis. These two signal regulation mechanisms help cells cope with ERS (Quiros et al., 2017). The PERK-ATF4-CHOP signaling pathway is involved in neuronal apoptosis (Gu et al., 2020). ATF4 can promote the expression of some genes that are conducive to cell survival, and this coordinated prosurvival response is called the integrated stress response (Young and Wek, 2016; Hetz and Saxena, 2017).

Studies have also shown that phosphorylated PERK/eIF2α is important for activation of ERS-related autophagy. Once eIF2α is phosphorylated, it can promote the conversion of microtubule-associated protein 1A light chain 3 (LC3)-I to LC3-II (Hoyer-Hansen and Jaattela, 2007). During autophagy, LC3-I is transformed into LC3-II by cleavage of amino acids at the hydroxyl end, which activates the autophagy system (Gao et al., 2013). In addition, a recent study showed that PERK signaling participates in oxygen-glucose deprivation/reoxygenation (OGD/R)-induced microglial activation and neuroinflammatory responses following PTP1B inhibitor treatment. After CIRI, the PERK pathway is activated, the expression of ERS marker proteins is increased, and autophagy is activated. In microglia, a PTP1B inhibitor alleviates the deleterious effects of CIRI and plays a neuroprotective role by inhibiting autophagy in rats (Zhu et al., 2021).

Protein kinase R-like endoplasmic reticulum kinase can not only regulate eIF2α but also phosphorylate nuclear factor erythroid 2-related factor 2 (NRF2). NRF2 is involved in the regulation of the cellular stress response and can induce the expression of antioxidant enzymes (Oh and Jun, 2017). Under physiological conditions, NRF2 binds to its negative regulator Kelch-like ECH-associated protein 1 (Keap1) (Hu L. et al., 2018). It is phosphorylated by PERK and dissociates from Keap1 under oxidative stress conditions before translocating into the nucleus where it activates the expression of NRF2-dependent antioxidant genes (Cullinan et al., 2003; Waza et al., 2018). Ultimately, it can reduce apoptosis during ERS and maintain the redox balance in cells (Cullinan and Diehl, 2004). Oxidative stress leads to NRF2 activation, which in turn inhibits the increase in ROS levels and ameliorates cellular damage caused by oxidative stress (Ramezani et al., 2018). Some studies have shown that the levels of NRF2 and HO-1 decrease significantly, indicating that NRF2/HO-1 signaling is involved in CIRI (Tian et al., 2020). Therefore, NRF2 is an important signaling factor related to the PERK signaling pathway, and its downstream signaling pathway should be further studied.

### The ATF6 Pathway

In mammals, ATF6 is an n-type membrane protein located in the ER (Haze et al., 1999). Its C-terminal end, which is inserted in the ER lumen, contains a GRP78-binding site and Golgi localization signal. The cytoplasmic N-terminal region contains basic leucine zipper (bZIP) and DNA transcriptional activity domains. ATF-6 has two configurations: ATF-6α and ATF-6β (Zhu et al., 1997). The former plays a leading role in ERS. When ERS occurs in cells, ATF6 is transported into the Golgi apparatus via the Golgi localization signal. Within the Golgi, ATF6 is cleaved by the serine proteases site 1 protease (S1P) and site 2 protease (S2P) to release the cytoplasmic fragment ATF6f, resulting in the activation of the protein (Ye et al., 2000). Activated sATF6 is a transcription factor containing a bZIP domain. After sATF6 leaves the Golgi apparatus and enters the nucleus, it combines with *cis*-acting endoplasmic reticulum stress response elements (ERSEs) in the nucleus to regulate and activate the expression of BiP, GRP94, and calnexin (Yoshida et al., 2001b; Wu et al., 2007; Yamamoto et al., 2007). In addition, ATF6 can stimulate the expression of CHOP and promote initiation of the UPR (Patwardhan et al., 2016).

Many studies have shown that an increase in ATF6 expression can regulate ERS and reduce cellular damage. A recent study showed that ischemic preconditioning can induce ATF6 expression, reduce ERS, and ultimately exert a neuroprotective effect (Lehotsky et al., 2009). Some studies have shown that the neurological function score of sATF6 knock-in mice is significantly increased, suggesting that activation of the ATF6 pathway can improve the outcome of CIRI (Yu et al., 2017). In addition, because the active form of ATF6 is rapidly degraded, the precursor of ATF6 can be used as a marker of early ERS (Thuerauf et al., 2002). Research has shown that ATF6α knockout mice exhibit more severe functional damage and a worse prognosis after myocardial ischemia or cerebral ischemia, indicating that ATF6 deficiency increases organ damage upon exposure to ischemia (Yoshikawa et al., 2015). Recent studies have shown that activation of the ATF6 signaling pathway in the brain after cardiac arrest is conducive to alleviating brain function impairment (Shen et al., 2021). Furthermore, a study showed that decreasing the cleavage of ATF6 in the Golgi apparatus can result in neuroprotection (Gharibani et al., 2013). It was also found that in a cerebral ischemia animal and reoxygenation cell models, taurine can inhibit the activation of ATF6, inhibit ERS, reduce cell apoptosis, and exert a neuroprotective effect after cerebral ischemia/reperfusion (Gharibani et al., 2013). Further molecular biology experiments are needed to validate the regulatory mechanism of ATF6 and its potential for CIRI treatment.

### The IRE1 Pathway

Inositol-requiring enzyme 1α is a type 1 transmembrane protein that contains an N-terminal domain, cytoplasmic C-terminal (RNase) domain, and serine/threonine kinase domain (Liu et al., 2003; Lee et al., 2008). There are two IRE1 isoforms in mammals: IRE1α, which is ubiquitously expressed, and IRE1β, which is mainly expressed in the gastrointestinal tract and pulmonary mucosal epithelium (Martino et al., 2013). Both of these isoforms are involved in signal transduction in ERS.

During ERS, unfolded proteins that accumulate in the ER bind GRP78. GRP78 then dissociates from IRE1, causing homodimerization and autophosphorylation of IRE1, which subsequently causes the activation of its RNase domain (Korennykh et al., 2009). Activated IRE1 can cleave XBP1 precursor mRNA, resulting in the generation of active spliced XBP1 (sXBP1) (Yoshida et al., 2001a), which is a bZIP transcription factor (Liou et al., 1990). After entering the nucleus, sXBP1 mRNA is translated to generate a mature protein which can promote the expression of protein folding-related genes and ultimately alleviate ERS (Travers et al., 2000; Chen and Brandizzi, 2013; Hetz and Saxena, 2017). Studies have shown that XBP1 is related to ER-mediated degradation of many components, and that its degradation ability is dependent on IRE1 (Yoshida et al., 2003). However, it should be noted that sXBP1 mRNA is quickly cleared from cells and is replaced by the uncleaved form (Marciniak et al., 2004). Studies have shown that under pathological conditions *in vitro*, ERS can cause complete cleavage of XBP1 mRNA. However, there have only been a few studies on this phenomenon. Therefore, care should be taken when performing quantitative analysis (Hosoi et al., 2010). sXBP1 is a key transcription factor in the regulation of cell survival (Hetz and Saxena, 2017). Persistent ERS results in sXBP1-mediated initiation of apoptosis via induction of CHOP expression (Dai et al., 2014).

Regarding UPR activation, celecoxib reduces ERS by promoting the IRE1-UPR pathway and ultimately exerts a neuroprotective effect (Santos-Galdiano et al., 2021). In addition, IRE1 contributes to ERS-mediated apoptosis through the c-Jun N-terminal kinase (JNK) and caspase-12 pathways. IRE1 can combine with TRAF2 to activate apoptosis signal-regulating kinase-1 (ASK1) and ultimately activate the JNK pathway and caspase-12, causing apoptosis (Nishitoh et al., 2002; Schonthal, 2013). It has been reported that taurine can significantly inhibit the IRE1 pathway and reduce apoptosis in animals and cell models (Gharibani et al., 2013). In addition, IRE1α can activate the NF-κB signaling pathway to initiate inflammatory reactions. In particular, the RNase domain of IRE1α mediates the degradation of a variety of mRNAs and microRNAs through a process called regulated IRE1-dependent decay (RIDD) and regulates pathological processes such as inflammation and apoptosis (Ghosh et al., 2014; Feldman et al., 2016; Wong et al., 2018).

## Endoplasmic Reticulum Stress and Cell Apoptosis

**FIGURE 2:**
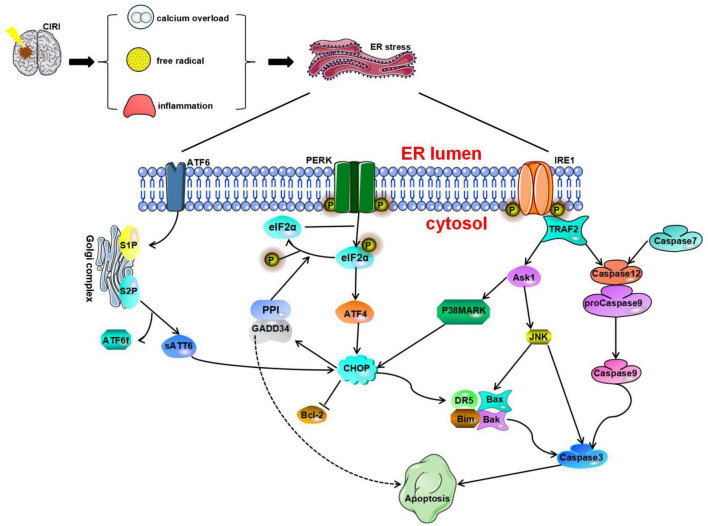
Endoplasmic reticulum stress is a harmful process that induces apoptosis mediated by CAAT/enhancer-binding protein (C/EBP) homologous protein (CHOP), caspase-12, and JNK. Glucose-regulated protein 78 (GRP78) dissociates from protein kinase R-like endoplasmic reticulum kinase (PERK), ATF6, and IRE1 and ultimately initiates proapoptotic signaling pathways by activating CHOP. All three pathways of the UPR can induce CHOP activation. Phosphorylated eukaryotic initiation factor 2α (IF2α) can promote ATF4 expression and then activate the expression of the downstream protein CHOP and induce cell apoptosis. Furthermore, the translation of CHOP is regulated by ATF6. CHOP can increase the expression of Bim, death receptor 5 (DR5), Bax, and Bak and inhibit the expression of Bcl-2 to play a proapoptotic role. The IRE1 pathway and caspase-7 pathway can cause activation of caspase-12. Activated caspase-12 promotes the activation of caspase-3/9 and eventually leads to apoptosis. TRAF2 recruits and activates ASK1, which subsequently phosphorylates and activates JNK.

Apoptosis is an important cell death pathway. Apoptosis is involved in the pathophysiological process of CIRI (Uzdensky, 2019). However, the process of neuronal apoptosis is complex. Recent studies have shown that three signal transduction pathways are involved in the regulation of apoptosis: the ERS pathway, the mitochondrial pathway, and the death receptor pathway (Ten and Galkin, 2019; Datta et al., 2020). ERS plays a vital role in stroke-induced neuronal apoptosis (Rao et al., 2004; Rozpedek et al., 2017; Mohammed et al., 2020). When cells cannot overcome external stress conditions, the UPR disrupts intracellular homeostasis and promotes apoptosis through CHOP/growth arrest, DNA damage-inducible gene 153 (GADD153), caspase-12, and JNK (Figure 2; Xin et al., 2014; Hetz et al., 2015; Hetz and Papa, 2018).

### CHOP Signaling

CAAT/enhancer-binding protein (C/EBP) homologous protein, also known as GADD153, is a member of the C/EBP transcription factor family, which some studies have proven to be an important executor of ERS-induced apoptosis (Huang et al., 2017; Hu H. et al., 2018).

Studies have shown that CHOP is involved in apoptosis in the nervous system (Wang et al., 2013; Xin et al., 2014). The process through which CHOP causes apoptosis is described as follows. After GRP78 dissociates from PERK, ATF6, and IRE1, it activates CHOP and the proapoptotic signaling pathway. All three pathways of the UPR can induce CHOP activation. After ERS, ATF4, cleaved ATF6α, and XBP1 undergo nuclear translocation, resulting in the rapid and significant upregulation of CHOP expression (Yang et al., 2017), phosphorylated eIF2α can promote the expression of the transcription factor ATF4. Under stress conditions, the ATF4 signaling pathway can regulate redox reactions, amino acid metabolism, autophagy, and apoptosis. During irreversible cell stress, ATF4 can also activate the expression of the downstream protein CHOP and initiate cell apoptosis. Studies have shown that canopy FGF signaling regulator 2 (CNPY2) is involved in the regulation of ERS (Hong et al., 2017). During ERS, the binding partner of CNPY2 changes from GRP78 to PERK, resulting in activation of the PERK-CHOP pathway and promotion of apoptosis (Urra and Hetz, 2017). Inhibition of the CNPY2 signaling pathway can block neuronal apoptosis induced by CIRI, leading to neuroprotection (Zhao et al., 2021). In addition, a study showed that the transcription of CHOP is regulated by ATF6 (Yoshida et al., 2000). Although moderate ERS helps promote the proper folding and modification of problem proteins, excessive or prolonged ERS may lead to activation of CHOP and caspase-3 signaling and promote apoptosis (Addinsall et al., 2018). Studies have shown that CHOP can increase the expression of the proapoptotic protein Bim and inhibit the expression of the antiapoptotic protein Bcl-2 to play a proapoptotic role (McCullough et al., 2001; Puthalakath et al., 2007; Logue et al., 2013). In a study on CIRI, Tajiri et al. (2004) found that the number of apoptotic neurons is significantly reduced in CHOP knockout mice and that CHOP is involved in the regulation of apoptosis and the expression of antiapoptotic Bcl-2 protein family members. Furthermore, CHOP can induce the expression of death receptor 5 (DR5), which makes cells more sensitive to ER-induced apoptosis, ultimately promoting apoptosis (Yamaguchi and Wang, 2004). Furthermore, after cerebral ischemia, Bax, BAD, and Bak can translocate from the cytoplasm to the outer mitochondrial membrane, resulting in the release of Cyt-c and activation of Caspase-3, resulting in apoptosis (Broughton et al., 2009). Another target gene of CHOP is GADD34. Induction of GADD34 expression can inhibit the downstream PERK signaling pathway. In addition, GADD34 is essential for regulation of protein synthesis during ERS (Walter and Ron, 2011) and can regulate the phosphorylation of eIF2α (Marciniak et al., 2004).

The expression of CHOP during CIRI may depend on the state of the cell and the intensity of ischemia (Osada et al., 2010). CHOP mainly promotes apoptosis during the early stage of reperfusion (Tajiri et al., 2004). A study showed that CHOP protein expression is increased 3 h after cerebral ischemia/reperfusion, peaks at 24 h, and begins to decrease at 48 h, which is consistent with the timeline of neuronal apoptosis. Furthermore, α-difluoromethylornithine (DFMO) treatment can inhibit ERS by inhibiting the expression of CHOP and exert a neuroprotective effect after ischemia/reperfusion (Ding and Ba, 2015). These results prove that drugs that regulate the expression of CHOP can affect the prognosis of CIRI and that CHOP is a potential target for the treatment of CIRI.

### Caspase-12 Signaling

Caspase-12 is a member of the IL-1β-converting enzyme (ICE) caspase subfamily. It is an important regulator of apoptosis and inflammation (García de la Cadena and Massieu, 2016). Caspase-12 usually negatively regulates the inflammatory response. It can inhibit the activation of caspase-1 in the inflammasome and regulate the expression of IL-1β and IL-18. Caspase-12 mRNA can be found in almost all tissues in mice. Under normal physiological conditions, TRAF2 forms a stable complex with procaspase-12. However, under stresses conditions, caspase-12 dissociates from TRAF2 (Yoneda et al., 2001).

It has been found that after alleviation of ischemia in tissues or cells, the levels of CHOP, Bax, activated caspase-12, and caspase-3 increase significantly, while the expression of Bcl-2 decreases (Guo et al., 2021). To date, three main pathways that can activate caspase-12 have been identified: the IRE1 pathway, the m-calpain pathway, and the caspase-7 pathway. IRE1α can trigger caspase-12 activation, while inactive pro-caspase-12 dissociates from the ER membrane and is then cleaved to trigger apoptosis (Sano and Reed, 2013; Yao et al., 2018). On the other hand, caspase-12 can be cleaved by other proteases, such as calpain and caspase-7 (Martinez et al., 2010; de la Cadena et al., 2014). In addition, in human neuroblastoma SK-N-SH cells incubated with thapsigargin (Tg) or Aβ, calpain inhibitors block the cleavage of caspase-12, indicating that calpain can reduce the expression level of caspase-12 (Matsuzaki et al., 2010). After ERS, caspase-7 translocates to the surface of the ER, forms a complex with caspase-12 and GRP78 on the surface of the ER, and mediates the cleavage of caspase-12 (Rao et al., 2001). Further studies have shown that activated caspase-12 is released into the cytoplasm and induces the activation of caspase-3/9 (Morishima et al., 2002; Datta et al., 2018; Rong et al., 2020). Studies have also shown that cells lacking caspase-12 are resistant to apoptosis elicited by ERS inducers such as tunicamycin (Tm), Tg, and brefeldin A (BFA) (Nakagawa et al., 2000). Shibata et al. reported that caspase-12 is cleaved from 5 to 23 h after reperfusion following 1 h of ischemia in transient middle cerebral artery occlusion (tMCAO) model mice (Shibata et al., 2003). It has also been shown that the expression of PERK and caspase-12 in hippocampal neurons increases rapidly under glucose deprivation. This suggests that glucose deprivation alone can lead to caspase-12-dependent neuronal apoptosis (de la Cadena et al., 2014). Some researchers have suggested that caspase-12 can promote the apoptosis of neuronal cells, mainly during continuous aggravation of reperfusion (Zhu et al., 2012). Selenoprotein K (SELENOK) gene knockout can significantly induce ERS and lead to neuronal apoptosis (Jia S. Z. et al., 2021). However, due to the low proteolytic activity of SELENOK and the lack of related studies, the role of SELENOK in ischemia-induced apoptosis is still controversial (García de la Cadena and Massieu, 2016).

### c-Jun N-Terminal Kinase Signaling

c-Jun N-terminal kinase plays an important role in the stress response, is involved in neuronal oxidative stress injury, and can mediate neuronal apoptosis (Ji et al., 2017). Like the CHOP and caspase-12 pathways, the JNK signaling pathway, which is activated during ERS, is considered an apoptosis-promoting pathway.

Phosphorylation of IRE1 in the cytoplasm stimulates the activation of TRAF2, which in turn phosphorylates and activates ASK1 and ultimately activates JNK (Cao and Kaufman, 2012). In addition, nervous system inflammation, ischemia, oxidative stress, and other stimuli can activate the expression of JNK. JNK regulates apoptosis by phosphorylating Stat3, p53, and Bcl-2 (Chen et al., 2003). JNK promotes Cyt c release and regulates caspase activation. Activation of the JNK signaling pathway during CIRI can lead to apoptosis of neuronal cells. It has been found that signals generated by the cytoplasmic kinase domain of IRE1 can regulate the JNK signaling pathway and may affect the regulation of apoptosis (Chakrabarti et al., 2011). Activated JNK can promote the expression of caspase-3 and other apoptosis-related genes and further initiate death receptor or mitochondrial pathways to induce apoptosis (Zhao et al., 2015). One study found that overexpression of aldehyde dehydrogenase 2 (ALDH2) can regulate JNK and caspase-3 activation and transcription in a model of cerebral ischemia, resulting in a significant reduction in mitochondrial-related apoptosis. These results suggest that ALDH2 may affect JNK-mediated mitochondrial apoptosis in ischemic stroke (Xia et al., 2020). It has been found that ischemic brain injury is often accompanied by increased apoptosis of nerve cells and that this cell apoptosis is obviously related to continuous activation of the IRE1α/TRAF2, JNK1/2, and p38 MAPK signaling pathways (Chen et al., 2015).

Drugs and compounds that regulate the JNK pathway, which reduce apoptosis and exerts neuroprotective effects, have been explored in several studies. In the early stage of CIRI, JNK inhibitors can reduce ERS and apoptosis and alleviate CIRI (Zhu et al., 2012). SP600125 is an effective JNK inhibitor that can ameliorate brain injury after CIRI (Khan et al., 2020). Traditional Chinese medicine plays a unique role in the treatment of cerebral ischemia injury, but the components of traditional Chinese medicine compounds are complex. Thus, some studies have focused on the effects of traditional Chinese medicine extracts. A recent study showed that senkyunolide I (SEI), an active constituent of the traditional Chinese medicine Ligusticum chuanxiong Hort. exerts a neuroprotective effect against glutamate-induced cell death. In addition, SEI can significantly inhibit the JNK/caspase-3 signaling pathway (Wang et al., 2021). JLX001 is a novel compound with a structure similar to that of cyclovirobuxine D (CVB-D). Some studies have proven that JLX001 exerts a neuroprotective effect against focal cerebral ischemic injury. Some researchers have studied the protective effects of JLX001 against CIRI and its antiapoptotic effects. The results showed that JLX001 can reduce neuronal apoptosis by inhibiting the JNK signaling pathway, thus exerting a protective effect against ischemia/reperfusion injury (Zhou et al., 2019). Another study showed that butylphthalide exerts an antiapoptotic effect after cerebral ischemic injury and that its effect is related to the regulation of the JNK/p38 MAPK signaling pathway (Bu et al., 2021).

## Oxidative Stress and Cerebral Ischemia/Reperfusion Injury

Oxidative stress is characterized by an imbalance between oxidation and antioxidation. Under physiological conditions, ROS and RNS are involved in regulating various redox processes in cells and maintaining homeostasis of the intracellular environment. An increase in free radical levels is the main cause of oxidative stress (Valko et al., 2007). Some exogenous agents can stimulate the production of intracellular free radicals, such as ROS. When the level of ROS exceeds the antioxidant capacity of the cell, oxidative stress impairs intracellular protein synthesis and ER homeostasis and affects the survival of cells (Zeeshan et al., 2016). Excess Ca^2+^ is a source of free radicals in cells. An increase in the Ca^2+^ concentration in neuronal cells leads to activation of neuronal nitric oxide synthase (nNOS), which causes an oxidative stress response, cell homeostasis disruption, or cell injury. Another source of oxygen free radicals is mitochondria (Kalogeris et al., 2014). After cerebral ischemia/reperfusion, activated microglia can promote the production of ROS (Zrzavy et al., 2018). Neurons have high metabolic activity, consume a large amount of oxygen, express relatively low levels of endogenous antioxidant enzymes (such as catalase), and are particularly sensitive to oxidative stress. Thus, oxidative stress can easily cause neuronal cell damage.

Although the pathophysiological mechanism of ischemic stroke is complex, oxidative stress may play a key role in injury caused by ischemic stroke (Manzanero et al., 2013). Moreover, an increasing number of researchers are paying attention to the mechanism by which oxidative stress leads to brain damage after CIRI (Lorenzano et al., 2018). As described earlier, when the levels of ROS and RNS exceed the capacity of the intracellular antioxidant system, oxidative stress and even cell damage occur. At low levels, ROS can act as signaling molecules in a variety of cellular processes (Scherz-Shouval and Elazar, 2007; Weidinger and Kozlov, 2015). ROS play a key role in the physiological regulation of metabolism and cell survival (Vicente-Gutierrez et al., 2019). However, when the level of ROS exceeds the capacity of the antioxidant and repair systems, ROS can oxidize various intracellular molecules or components, including lipids, DNA, proteins, and mitochondria, causing cell damage. Excessive production of ROS is considered an important mechanism underlying neuronal injury in the brain and impairment of nervous system function during CIRI (Ding et al., 2014). Excessive production of ROS affects the homeostasis of the intracellular environment, damages the normal structure of cells, and affects cell function, ultimately leading to cell necrosis and apoptosis (Cobley et al., 2018). These findings provide a direction for the development of treatments for ischemic stroke. Particularly, some researchers consider redox homeostasis maintenance a method for ischemic stroke treatment.

During reperfusion, the recovery of cerebral blood flow increases the amount of oxygen and nutrients supplied to brain tissue, which is very important for improving cell survival. However, this oxygen may also be used by pro-oxidant enzymes and mitochondria to produce excessive ROS in neuronal tissue, thus contributing to new and exacerbated tissue damage (Chen et al., 2011). This further proves that oxidative stress plays an important role in cerebral ischemic injury. Other studies have shown that oxidative stress can induce the release of Cyt c, leading to mitochondrial dysfunction, alterations in cell energy sources, and, ultimately, apoptosis (Chen et al., 2011). Regarding the specific mechanism, it has been found that cerebral ischemia leads to depolarization of the mitochondrial membrane potential (ΔΨm), a reduction in ATP production, extracellular calcium overload, and the release of Cyt c, eventually leading to neuronal death (Liu et al., 2018b; Salehpour et al., 2019). During cerebral ischemia/reperfusion, a large amount of ROS is produced in mitochondria, and these ROS are transported to the outer mitochondrial membrane by Bcl-2 and the proapoptotic protein Bax. These then polymerize to form a membrane channel, which promotes the release of Cyt c from mitochondria into the cytoplasm. Cyt c released into the cytoplasm binds Apaf-1, combines with caspase-9 to form a complex, and finally activates caspase-3. Activated caspase-3 can cleave many nuclear DNA repair enzymes, preventing the repair of nuclear DNA damage during cerebral ischemia and causing apoptosis. CIRI causes mitochondrial edema and fragmentation, further inhibits the synthesis of ATP, and increases the production of ROS, directly leading to necrotic cell death. It has been found that at physiological concentrations, ROS coordinate with the antioxidant system *in vivo* and maintain cell function and the redox state. However, at high concentrations, ROS can inhibit the body’s antioxidant defense system (Dasuri et al., 2013). Therefore, after a large amount of ROS passes through the ER membrane, which contains a large amount of lipids, ER function may be further impaired. In addition, oxidative stress can promote the formation of abnormal sulfur bonds, cause the production of a large number of abnormal intermediates, and inhibit the degradation of misfolded proteins.

Oxidative stress and inflammation interact during cerebral ischemia. Adaptive protection of the body during cerebral ischemia stimulates aseptic inflammation in the ischemic area. However, during reperfusion, ROS and blood-derived anti-inflammatory factors enter the ischemic tissue and the surrounding area, aggravating the inflammatory reaction. Furthermore, studies have shown that the UPR can trigger inflammation through its interaction with NF-κB. In turn, inflammation aggravates dysfunction of the internal environment, which can further aggravate ERS (Chaudhari et al., 2014). If this inflammatory response is not alleviated, various factors can trigger the apoptosis pathway mediated by the ER and mitochondria; that is, they can activate caspase-1 and caspase-9, further activate caspase-3 and deoxyribonucleases, induce DNA breaks, activate caspase-12 on the ER, and ultimately mediate apoptosis (Raturi and Simmen, 2013; Ye et al., 2013). Excess ROS may also damage endothelial cells (ECs) and degrade tight junction (TJ) proteins, greatly increasing the permeability of the blood–brain barrier (BBB). As a result, exogenous macromolecules can easily cross the BBB and enter brain tissue, further aggravating brain tissue injury and affecting neuronal function (Zhang et al., 2017). Ischemia/reperfusion facilitates the inflammatory response mediated by oxidative stress in ECs and promotes the recruitment and infiltration of peripheral immune cells into the ischemic area. The accumulation of immune cells and proinflammatory cytokines further promotes BBB disruption and aggravates brain injury (Jin et al., 2019).

Increasing evidence indicates that strategies that eliminate excess free radicals are beneficial in some diseases. Because oxidative stress is the key factor in BBB disruption and neuroinflammation, reducing the production of ROS in cells is a potential strategy for treating cerebral ischemia. Studies have found that some drugs, such as hesperidin, apigenin, and diosmin, can reduce the production of ROS, alleviate brain edema, decrease leukocyte aggregation in the ischemic area, and exert a protective effect against reperfusion injury (Mastantuono et al., 2015). Peroxiredoxin 4 (Prx4), a member of the antioxidant enzyme family (Prx1–6), is an efficient H_2_O_2_ scavenger. Within the ER, Prx4 can effectively eliminate peroxides (Zhu et al., 2014). Antioxidants can inhibit the production of intracellular ROS, attenuate damage to the BBB, and ameliorate brain injury (Zhang et al., 2017). Therefore, Prx4 may protect neuron function and alleviate CIRI by protecting EC function, reducing BBB damage, and regulating the inflammatory response (Yang et al., 2021). Mitochondria are the main organelles involved in regulation of cellular ROS production (Kausar et al., 2018). In line with this, studies have shown that natural and synthetic polyphenols increase the expression of antioxidant enzymes and cell protective proteins, reduce oxidative stress, inhibit the cellular inflammatory response, and protect cell function (Duong et al., 2014). In addition, these compounds can enhance mitochondrial function and biogenesis (Chen et al., 2019).

Endoplasmic reticulum stress and oxidative stress interact closely. An increase in the amount of unfolded proteins in the lumen of the ER can lead to the release of a large amount of calcium from the ER, and entry of calcium into mitochondria can impair the function of mitochondria, lead to the production of excessive ROS, and promote oxidative stress (Zhang et al., 2016). Furthermore, oxidative stress is an important cause of ERS (Nakka et al., 2016). When cells undergo oxidative stress, the redox balance of the ER is disruption, leading to impairment of ER function and ERS. Therefore, ERS and oxidative stress are closely related and should not be studied in isolation. We look forward to new research on their interaction.

## Cross-Talk Between Endoplasmic Reticulum Stress and Mitochondria

The mitochondria generates ATP, contributes to Ca^2+^ homeostasis, and regulation of ROS production. Mitochondrial dysfunction can impair cell energy production and cause oxidative stress, cellular injury, or apoptosis. Furthermore, mitochondrial dysfunction is an important factor in CIRI. In ischemic stroke, local cerebral blood flow is blocked, the supply of nutrients and oxygen is reduced, and the production of ATP is impaired, resulting in cell death. Mitochondrial dysfunction impairs energy generation, increases ROS production, and stimulates Cyt c release into the cytosol (Giorgi et al., 2018). Cells respond to environmental changes through autophagy. As a defense mechanism, autophagy can remove damaged organelles and metabolites in cells. Mitophagy is a selective form of macroautophagy. Its main function is to eliminate superfluous or damaged mitochondria and maintain normal cell function. In recent years, studies have shown that mitophagy can alleviate CIRI and play a neuroprotective role through a variety of mechanisms. However, the role of mitophagy in CIRI remains controversial. Some experts believe that excessive mitophagy can lead to cell death.

The ER is structurally and functionally coupled to mitochondria. In the axons of rodents, approximately 5% of the mitochondrial surface contacts the ER, forming an interconnected network which is conducive to the direct transport of Ca2^+^ from the ER to mitochondria (Wu et al., 2017b). The endoplasmic reticulum-mitochondria encounter structure (ERMES) forms a junction between the mitochondria and the ER, which is involved in maintaining the morphological structure and function of the ER and mitochondria. Four ERMS proteins have been found in yeast, including the ER-anchored protein Mmm1 and three mitochondrial-related proteins, i.e., Mdm10, Mdm12, and Mdm34. Their functions are related to mitochondrial morphology and protein production (Stroud et al., 2011). In addition, the ER and mitochondria are both tubular organelles with dynamic characteristics. Thus, there are many contact points between them, and they interact to form regional membranes, namely, MAMs (Giacomello et al., 2020). MAMs are rich in glycosphingolipid-enriched microdomains (GEMs), which are structures that control Ca2^+^ flow between the ER and mitochondria. In addition, MAMs can regulate lipid metabolism and the inflammatory response (Raturi and Simmen, 2013; Marchi et al., 2014). Inositol 1,4,5-trisphosphate receptors (IP3Rs) are the principal Ca^2+^ channels that regulate Ca^2+^ flux in these regions (Ahumada-Castro et al., 2021). Regarding the transfer of Ca^2+^, it has been found that the voltage-dependent anion channels (VDACs) help Ca^2+^ released from the ER enter mitochondria (Csordas et al., 2018). IP3R is involved in mediating the release of Ca^2+^ into mitochondria, where Ca^2+^ regulates the activity of several enzymes and transporters.

There is also functional coupling between the ER and mitochondria, and they interact and depend on each other (Giorgi et al., 2009). ATP produced by mitochondrial oxidative phosphorylation is the energy source for correct folding of ER proteins. In addition, lipids produced during the folding of ER proteins are the material basis for the stability of the mitochondrial membrane. As the storage site for neutral lipids, lipid droplets (LDs) play a central role in FA homeostasis. LDs mainly contact the ER, but also contact mitochondria, peroxisomes, and lysosomes (Valm et al., 2017; Shai et al., 2018). Contacts between LDs and these organelles contribute to the maintenance of energy balance and cell survival. In addition, LD and organelles interact to form a metabolic center and regulate the biogenesis, growth, and distribution of LDs (Hariri et al., 2018; Ugrankar et al., 2019; Henne et al., 2020). Therefore, abnormal protein translation at LD contacts leads to various metabolic disorders (Herker et al., 2021).

Moreover, studies have shown that Ca^2+^ underlies the functional coupling between the ER and mitochondria and that Ca^2+^ transport from the ER to mitochondria plays an important role in regulating cell bioenergy, ROS production, autophagy, and apoptosis (Kaufman and Malhotra, 2014). In fact, the regulation of mitochondrial function is closely related to Ca^2+^ (Glancy and Balaban, 2012), and mitochondrial energy balance is regulated by Ca^2+^(Bustos et al., 2017). Studies have shown that Ca^2+^ levels fluctuate during the cell cycle (Humeau et al., 2018); however, recently, Koval et al. (2019) found that uptake of Ca^2+^ in mitochondria through the mitochondrial Ca^2+^ uniporter (MCU) is necessary for to the production of energy by mitochondria and the maintenance of cell function. After cerebral ischemia, intracellular H^+^ levels increase, and the pH decreases due to anaerobic metabolism. To improve the intracellular environment, the intracellular ion exchange system is activated, resulting in intracellular calcium ion overload. Due to a rapid decrease in ATP content, the function of calcium ion pumps on the ER membrane, such as SERCA pumps, is impaired, and calcium cannot be absorbed. However, calcium ions stored in the ER are released into the cytoplasm, further aggravating intracellular calcium overload, disrupting calcium homeostasis in the ER and triggering or aggravating ERS.

We believe that ERS and mitophagy regulate each other, and that they are involved in the regulation of intracellular homeostasis. At present, the specific mechanisms underlying the interaction between ERS and mitochondria in ischemic stroke are not completely clear. Therefore, further detailed studies are needed to reveal the complex interaction network between them to provide a theoretical basis for improving CIRI treatments.

## Endoplasmic Reticulum Stress Plays an Important Role in Cerebral Ischemia/Reperfusion Injury

Endoplasmic reticulum stress is one of the mechanisms involved in CIRI. It may also play different roles in different stages of CIRI. The initial purpose of ERS is to maintain ER homeostasis, but prolonged or severe ERS may be harmful (Martin-Jimenez et al., 2017). Studies have shown that the UPR can promote the degradation of unfolded or incorrectly folded proteins and protect cells in the early stage of ischemia (Xin et al., 2014). However, upon prolongation of ischemia, the UPR can promote apoptosis. ERS and ERS-related apoptosis have been reported to contribute to ischemia/reperfusion injury (Wu et al., 2017a). The ER is sensitive to ischemia. Particularly, ER homeostasis is disrupted by hypoxia-hypoglycemia beginning in the early ischemic period, and ERS and ERS-related apoptosis are triggered and exacerbated in the reperfusion period (Wu F. et al., 2018). Hence, the mitochondrial pathway, the death receptor pathway, and ERS are generally considered the three primary apoptotic pathways (Gillies and Kuwana, 2014).

It has been demonstrated that regulation of the ERS-related signaling pathway is protective during ischemic stroke. Likewise, the PERK pathway may play a protective role in the early stage of ischemic stroke. Yoshikawa et al. (2015) showed that the ATF6 pathway participates in the early stage of ischemia, promotes the survival of neurons and glial cells, and plays a protective role in ischemic stroke. Gharibani et al. (2013) also proved that XBP1 might play a protective role by increasing the Bcl-2/Bax ratio and downregulating Caspase-3 expression *in vitro* during ischemia/reperfusion injury. These findings provide a theoretical basis for the development of related drugs for the treatment of CIRI via regulation of ERS.

Many studies have shown that the UPR can promote apoptosis in the late stage of ischemic stroke and that CHOP, Caspase-12, and JNK are involved in this process, with CHOP playing a leading role (Lopez-Hernandez et al., 2015; Poone et al., 2015). There is further evidence that the PERK pathway plays a major role in the expression of CHOP (Mei et al., 2013; Xin et al., 2014). A study showed that ERS promotes apoptosis through the PERK/eIF2α/caspase-3 pathway and that atorvastatin can reduce the protein expression of PERK, the dephosphorylation of eIF2α, and the activity of caspase-3, thus alleviating CIRI (Yang and Hu, 2015). In particular, excessive ERS can alter the permeability of the BBB (Kwak et al., 2015; Qie et al., 2017; Xu et al., 2019), making it easy for harmful substances to enter brain tissue. A recent study showed that salvinorin A can inhibit the ERS response, inhibit the production of ROS, reduce human brain microvascular endothelial cell (HBMEC) apoptosis, and increase the permeability of the BBB, ultimately alleviating brain injury and protecting neuronal function by activating the AMPK signaling pathway (Xin et al., 2021). Another study showed that adenosine acts as an endogenous neuroprotector by regulating Ca^2+^ homeostasis and glutamate release, reducing excitotoxic cellular damage after cerebral ischemia/reperfusion (Martire et al., 2019).

It has been found that inhibiting ERS can ameliorate CIRI and protect neuronal function (Nakka et al., 2010). Furthermore, studies have shown that hypoxia/reoxygenation (H/R) can induce ERS, increase the expression of ATF6 and GRP78, and ultimately lead to apoptosis. Liquiritin (LQ) treatment can reduce the expression of ATF6 and GRP78, inhibit the ERS pathway, and reduce apoptosis (Li et al., 2021). A study showed that the combination of *S*-methyl-*N,N*-diethyldithiocarbamate sulfoxide (DETC-MeSO) and taurine can reduce ERS in the ipsilateral ischemic penumbra; inhibit the ATF6, PERK, and IRE1 pathways; and reduce apoptosis (Gharibani et al., 2015). It has been found that lncRNAs are closely related to human diseases. Furthermore, some studies have shown that lncRNAs are involved in CIRI. It was found that the expression of MALAT1 is significantly increased during reperfusion in an OGD/R cell model. MALAT1 silencing can inhibit ERS and neuronal apoptosis and reduce neuronal damage (Jia Y. et al., 2021). Nucleotide-binding oligomerization domain 1 (NOD1) activates autophagy and ERS, decreasing cell survival. This suggests that NOD1 ultimately leads to CIRI via activation of ERS-mediated autophagy. Conversely, downregulating the expression of NOD1 can inhibit ERS and increase the viability of cortical neurons (Ma et al., 2020). Hyperhomocysteinemia (HHcy) is a well-known risk factor for stroke. The UPR is activated in a diet-induced HHcy model, and vitamin B supplementation alleviates ERS. HHcy exacerbated cellular injury during OGD/R. These effects can be prevented by vitamin B cotreatment (Tripathi et al., 2016).

In recent years, there have been many studies on CIRI, and it has been found that CIRI can be alleviated via regulation of ERS. Some of these studies are listed (Table 1) below. In some studies, cell survival was improved by targeting the apoptosis pathway related to ERS. Therefore, it is necessary to study the role and mechanism of ERS.

**TABLE 1:** Several cytokines/compounds exacerbate or mitigate cerebral ischemia/reperfusion injury (CIRI) by regulating endoplasmic reticulum stress (ERS).

Cytokine/compound	Animal/cell model(s)	Model(s)	Intervention	Related protein changes	ERS pathway(s)	Effect	References
Taurine	Adult male Sprague–Dawley rats and primary cortical neurons	tMCAO and OGD/R	Taurine	Reduction in ERS (cleaved ATF6 and p-IRE1 levels) and decrease in apoptosis (caspase-3, CHOP, caspase-12, and BCL-2/Bax levels)	The ATF6 and IRE1 pathways	Protective	Gharibani et al., 2013
DETC-MeSO	Adult male Sprague–Dawley rats and primary neurons	tMCAO and OGD/R	DETC-MeSO	Reduction in ERS (p-PERK, p-eIF2α, ATF4, JNK, XBP-1, GADD34, and CHOP levels) and decrease in apoptosis (Bak, Bax, Bad, caspase-3, and BCL-2 levels)	The PERK pathway	Protective	Mohammad-Gharibani et al., 2014
Apelin-13	Adult male Wistar rats and primary cortical neurons	tMCAO and OGD/R	Apelin-13	Reduction in ERS (p-eIF2α, ATF4, CHOP and GRP78 levels) decrease in neuronal apoptosis	The PERK pathway	Protective	Wu F. et al., 2018
Aniline-derived compound (Comp-AD)	Male C57BL/6J mice	tMCAO	Comp-AD	Reduction in ERS (p-PERK and p-IRE1α levels)	The PERK and IRE1 pathways	Protective	Morihara et al., 2018
Dexmedetomidine	Male Sprague–Dawley rats and primary cortical neurons	tMCAO and OGD/R	Dexmedetomidine	Reduction in ERS (GRP78 and p-PERK levels) and decrease in apoptosis (CHOP, Caspase-11 and cleaved Caspase-3 levels)	The PERK pathway	Protective	Liu et al., 2018a
Homer1a	Primary cortical neurons	OGD/R	Homer1a overexpression	Reduction in ERS (p-PERK/PERK and p-IRE1/IRE1 levels) and alleviation of mitochondria dysfunction	The PERK pathway	Protective	Wei et al., 2019
Vascular endothelial growth factor (VEGF) inhibitor	Adult male BALB/C mice and BEND3 microvascular ECs	tMCAO and OGD/R	Intraperitoneal injection of anti-VEGF 30 min before MCAO and transfection with siRNA-VEGF	Reduction in ERS (XBP-1 and GRP78 levels), decrease in apoptosis (cleaved Caspase-3, CHOP and IRE1α levels), and decrease in ROS levels	The IRE1 pathway	Harmful	Feng et al., 2019
RTN1-C	N2a cells and primary neurons	OGD/R	RTN1-C knockdown	Reduction in ERS (GRP78, cleaved caspase-12, CHOP and cleaved caspase-3 levels) and decreases in cell viability and apoptosis	The PERK and IRE1 pathways	Harmful	Lin et al., 2019
Urolithin A	Male C57BL/6 mice, N2a cells and primary neurons	tMCAO and OGD/R	Intraperitoneal injection of Uro-A 24 h and 1 h before surgery	Alleviation of ERS (decreases in ATF6 and CHOP levels) and increase in cell viability	The PERK and ATF6 pathways	Protective	Ahsan et al., 2019
Icariin (ICA)	Primary microglia and primary cortical neurons	OGD/R	ICA	Alleviation of ERS (decreases in p-IRE1α, IRE1α, XBP1u, XBP1 s and Cleaved caspase-3 levels), enhancement of cell viability, and reduction in apoptosis	The IRE1 pathway	Protective	Mo et al., 2020
(–)-Clausenamide	Rat primary cortical neurons	OGD/R	(–)-Clausenamide	Inhibition of ERS (decreases in GRP78, eIF2α, ATF4 and CHOP levels) and attenuation of apoptosis (decrease in cleaved caspase-3 levels)	The PERK pathway	Protective	Wu et al., 2020
Ginsenoside Rg1	Sprague–Dawley rats and primary cortical neurons	tMCAO and OGD/R	Intraperitoneal injection of Rg1	Alleviation of ERS (decreases in PERK, eIF2α, ATF4, CHOP and TRB3 levels), inhibition of neuronal apoptosis (decreases in Bax, caspase-3, and BCL-2 levels), and improvement in neuronal viability	The PERK pathway	Protective	Gu et al., 2020
sc-222227	Male Wistar rats	tMCAO	Intracerebroventricular injection of sc-222227	Attenuation of ERS (decreases in p-PERK/total PERK, p-IRE1/total IRE1, and cleaved AFT6/full-length ATF6 levels)	The PERK, IRE1 and ATF6 pathways	Protective	Zhu et al., 2021
Berberine	PC12 cells	OGD/R	Berberine	Decrease in ERS (GRP78, CHOP, Bax and cleaved caspase-3 levels)	The PERK and IRE1 pathways	Protective	Xie et al., 2020
Cilostazol	Male Sprague–Dawley rats and brain microvascular endothelial cells (BMVECs)	tMCAO and OGD/R	Cilostazol	Attenuation of ERS (decreases in p-PERK, PERK, p-IRE1-α, IRE1-α, ATF-6, Bip levels)	The PERK, IRE1 and ATF6 pathways	Protective	Nan et al., 2019
CASP8 and FADD-like apoptosis regulator (CFLAR)	Male C57BL6 mice and primary human brain microvascular endothelial cells (HBMVECs)	tMCAO and OGD/R	CFLAR transfection and knockdown	Alleviation of ERS (decreases in GRP78, PERK, ATF6 and cleaved Caspase-12 levels) and increase in cell viability by CFLAR overexpression and reduction in cell viability by CFLAR silencing	The PERK and ATF6 pathways	Protective	Wang et al., 2019

## Concluding Remarks

After stroke, the degree of functional damage to nerve cells depends on the degree of tissue hypoperfusion. In the ischemic core, necrotic cells die within a few minutes. However, around the core necrotic area, there is often an ischemic marginal area, namely, the ischemic penumbra. Delayed cell death occurs through apoptosis. The goal of CIRI treatment is to preserve neurons in the ischemic penumbra and restore neuronal function as much as possible. The pathophysiological process of CIRI can trigger ERS, and ERS contributes to the occurrence and development of CIRI. In the present article, we describe various causes of ERS induced by CIRI, including calcium overload, ROS accumulation, and the inflammatory response. These factors not only lead to secondary brain injury but also hinder the recovery of neurological function after treatment. The degree of ERS determines the fate of cells. In the early stage of cerebral ischemia/reperfusion, ERS can relieve damage to the ER and promote cell survival by initiating the UPR. In this paper, we also discussed the three signal transduction pathways related to ERS in detail. Excessive ERS leads to apoptosis, aggravates CIRI, and promotes apoptosis through the CHOP, caspase-12, and JNK signaling pathways. We also discussed the regulatory mechanism of these three signaling pathways in detail.

In the future, more in-depth research is needed to elucidate the specific mechanism underlying these phenomena. For example, when does ERS protect against and exacerbate ischemic stroke? Is the role of ERS different in different kinds of cells? In addition to considering the mechanisms and treatment effects in neurons, we should also pay attention to other cell populations, such as microglia. Animal studies have proven that inhibiting ERS can reduce the volume of the cerebral infarct, but how far are ERS inhibitors from clinical application? It is important to further determine the interaction between ERS and apoptosis and between ERS and inflammation to identify effective biological strategies for alleviating ERS and preventing brain injury after stroke. A large number of studies on the potential of alleviating CIRI using strategies targeting the apoptosis and inflammation pathways have been carried out, but more research, drug development, and clinical trials are needed. In addition, there are many studies on the molecular mechanism of ERS, but there have been few studies on the interaction between ERS, oxidative stress, and mitochondrial dysfunction. We believe that the interaction between these processes is worthy of in-depth study. At present, it is believed that interventions targeting ERS, including those that alter the expression of ligands in the ERS pathway and their receptors, can ameliorate CIRI and protect neuronal function. In addition, preventing the occurrence and development of brain cell apoptosis induced by ERS, which can protect neuronal function, may alleviate CIRI. We believe that solving these problems will open a new chapter in the treatment of ischemic stroke. Targeting ERS to treat CIRI is an important research direction. There are many mechanisms and answers that are not clear. Future research should focus on solving these problems and translating potential treatments from the laboratory to the clinic as soon as possible. ERS-targeted therapeutic strategies for cerebral ischemia are exciting areas of research as there are many unanswered questions. More careful research is needed in the future to translate such therapies from the laboratory to the clinic. In addition, previous studies focused on individual mechanisms underlying cerebral ischemic injury. We believe that these mechanisms occur simultaneously and synergize after cerebral ischemia. Therefore, we should study them as a whole and pay attention to their interaction.

## Author Contributions

LW wrote the initial draft. YL contributed to reviewing the literature. XZ and YY prepared the figures and submitted the manuscript. XX and SZ collected the literature and made the tables. ZJ, LG, and HW designed the manuscript and prepared the final version. All authors read and approved the final manuscript.

## Conflict of Interest

The authors declare that the research was conducted in the absence of any commercial or financial relationships that could be construed as a potential conflict of interest.

## Publisher’s Note

All claims expressed in this article are solely those of the authors and do not necessarily represent those of their affiliated organizations, or those of the publisher, the editors and the reviewers. Any product that may be evaluated in this article, or claim that may be made by its manufacturer, is not guaranteed or endorsed by the publisher.

## Glossary

ER, endoplasmic reticulum; ER stress, endoplasmic reticulum stress; CIRI, cerebral ischemia-reperfusion injury; rtPA, recombinant tissue plasminogen activator; ICH, intracranial hemorrhage; ROS, reactive oxygen species; RNS, reactive nitrogen species; RNOS, reactive/nitrogen oxide species; UPR, unfolded protein response; EOR, ER over-load response; SREBP, sterol regulatory element binding protein; GRP78, glucose-related protein 78; ERAD, ER-associated degradation; UPS, ubiquitin proteasome system; MAMs, mitochondria-associated endoplasmic reticulum membranes; PM, plasma membrane; GRPs, glucose-regulated proteins; SERCA, sarco/endoplasmic reticulum Ca^2 +^ -ATPase; MPT, mitochondrial permeability transition; NOX, NADPH oxidase; XO, xanthine oxidase; MAO, monoamine oxidase; Cyt *c*, cytochrome *c*; MCAO, middle cerebral artery occlusion; IL-1 β, interleukin-1 β; IL-6, interleukin-6; TNF-α, tumor necrosis factor-α; NF-κB, nuclear factor κB; eIF2α, eukaryotic initiation factor 2α; IκB, inhibitors of nuclear factor κB; PGRN, progranulin; NLRP3, NLR family, pyrin domain-containing 3; PERK, protein kinase RNA-like endoplasmic reticulum kinase; IRE-1, inositol-requiring protein 1; ATF6, activating transcription factor 6; GRP78, glucose regulated protein 78; XBP1, X-box binding protein 1; NRF2, nuclear factor erythroid 2-related factor 2; GADD34, growth arrest and DNA damage 34; CHOP, CAAT/enhancer-binding protein (C/EBP) homologous protein; ATF4, activating transcription factor 4; TRB3, tribbles-related protein 3; OGD/R, oxygen-glucose deprivation/reoxygenation; PTP1B, protein tyrosine phosphatase 1B; Keap1, Kelch-like ECH associating protein 1; S1P, site 1 protease; S2P, site 2 protease; b ZIP, basic leucine zipper; JNK, c-Jun N-terminal kinase; ASK1, apoptosis signal-regulating kinase-1; TRAF2, tumor necrosis factor receptor-associated factor 2; RIDD, Regulated IRE1-Dependent Decay; DR5, death receptor 5; MPTP, mitochondrial permeability transition pore; OMM, outer mitochondrial membrane; LDs, lipid Droplets; DETC-MeSO, S-Methyl-*N*, *N*-diethyldithiocarbamate sulfoxide; NOD1, nucleotide-binding oligomerization domain 1.

## References

[B1] Addinsall A. B. Wright C. R. Andrikopoulos S. Poel C. V. Stupka N. (2018). Emerging roles of endoplasmic reticulum-resident selenoproteins in the regulation of cellular stress responses and the implications for metabolic disease. *Biochem. J.* 475 1037–1057. 10.1042/bcj20170920 29559580

[B2] Adibhatla R. M. Hatcher J. F. (2010). Lipid oxidation and peroxidation in CNS health and disease: from molecular mechanisms to therapeutic opportunities. *Antioxid. Redox Signal.* 12 125–169. 10.1089/ars.2009.2668 19624272

[B3] Adolph T. E. Tomczak M. F. Niederreiter L. Ko H. J. Bock J. Martinez-Naves E. (2013). Paneth cells as a site of origin for intestinal inflammation. *Nature* 503 272–276. 10.1038/nature12599 24089213 PMC3862182

[B4] Ahsan A. Zheng Y. R. Wu X. L. Tang W. D. Liu M. R. Ma S. J. (2019). Urolithin A-activated autophagy but not mitophagy protects against ischemic neuronal injury by inhibiting ER stress in vitro and in vivo. *CNS Neurosci. Ther.* 25 976–986. 10.1111/cns.13136 30972969 PMC6698978

[B5] Ahumada-Castro U. Bustos G. Silva-Pavez E. Puebla-Huerta A. Lovy A. Cárdenas C. (2021). In the right place at the right time: regulation of cell metabolism by IP3R-mediated inter-organelle Ca^2+^ fluxes. *Front. Cell Dev. Biol.* 9:629522. 10.3389/fcell.2021.629522 33738285 PMC7960657

[B6] Almanza A. Carlesso A. Chintha C. Creedican S. Doultsinos D. Leuzzi B. (2019). Endoplasmic reticulum stress signalling – from basic mechanisms to clinical applications. *FEBS J.* 286 241–278. 10.1111/febs.14608 30027602 PMC7379631

[B7] Baines C. P. (2009). The mitochondrial permeability transition pore and ischemia-reperfusion injury. *Basic Res. Cardiol.* 104 181–188. 10.1007/s00395-009-0004-8 19242640 PMC2671061

[B8] Bellezza I. Grottelli S. Mierla A. L. Cacciatore I. Fornasari E. Roscini L. (2014). Neuroinflammation and endoplasmic reticulum stress are coregulated by cyclo(His-Pro) to prevent LPS neurotoxicity. *Int. J. Biochem. Cell Biol.* 51 159–169. 10.1016/j.biocel.2014.03.023 24699213

[B9] Bertolotti A. Zhang Y. Hendershot L. M. Harding H. P. Ron D. (2000). Dynamic interaction of BiP and ER stress transducers in the unfolded-protein response. *Nat. Cell Biol.* 2 326–332. 10.1038/35014014 10854322

[B10] Blais J. D. Filipenko V. Bi M. X. Harding H. P. Ron D. Koumenis C. (2004). Activating transcription factor 4 is translationally regulated by hypoxic stress. *Mol. Cell Biol.* 24 7469–7482. 10.1128/mcb.24.17.7469-7482.2004 15314157 PMC506979

[B11] Broughton B. R. Reutens D. C. Sobey C. G. (2009). Apoptotic mechanisms after cerebral ischemia. *Stroke* 40 e331–e339. 10.1161/strokeaha.108.531632 19182083

[B12] Bu X. Y. Xia W. Q. Wang X. N. Lu S. Gao Y. (2021). Butylphthalide inhibits nerve cell apoptosis in cerebral infarction rats via the JNK/p38 MAPK signaling pathway. *Exp. Ther. Med.* 21:565. 10.3892/etm.2021.9997 33850537 PMC8027748

[B13] Bustos G. Cruz P. Lovy A. Cárdenas C. (2017). Endoplasmic reticulum-mitochondria calcium communication and the regulation of mitochondrial metabolism in cancer: a novel potential target. *Front. Oncol.* 7:199. 10.3389/fonc.2017.00199 28944215 PMC5596064

[B14] Campbell B. C. V. de Silva D. A. Macleod M. R. Coutts S. B. Schwamm L. H. Davis S. M. (2019). Ischaemic stroke. *Nat. Rev. Dis. Primers* 5:70. 10.1038/s41572-019-0118-8 31601801

[B15] Cao S. Kaufman R. (2012). Unfolded protein response. *Curr. Biol.* 22 R622–R626. 10.1016/j.cub.2012.07.004 22917505

[B16] Cao S. S. Kaufman R. J. (2014). Endoplasmic reticulum stress and oxidative stress in cell fate decision and human disease. *Antioxid. Redox Signal.* 21 396–413. 10.1089/ars.2014.5851 24702237 PMC4076992

[B17] Chakrabarti A. Chen A. W. Varner J. D. (2011). A review of the mammalian unfolded protein response. *Biotechnol. Bioeng.* 108 2777–2793. 10.1002/bit.23282 21809331 PMC3193940

[B18] Chaudhari N. Talwar P. Parimisetty A. Lefebvre d’Hellencourt C. Ravanan P. (2014). A molecular web: endoplasmic reticulum stress, inflammation, and oxidative stress. *Front. Cell. Neurosci.* 8:213. 10.3389/fncel.2014.00213 25120434 PMC4114208

[B19] Chen H. Yoshioka H. Kim G. S. Jung J. E. Okami N. Sakata H. (2011). Oxidative stress in ischemic brain damage: mechanisms of cell death and potential molecular targets for neuroprotection. *Antioxid. Redox Signal.* 14 1505–1517. 10.1089/ars.2010.3576 20812869 PMC3061196

[B20] Chen J. H. Kuo H. C. Lee K. F. Tsai T. H. (2015). Global proteomic analysis of brain tissues in transient ischemia brain damage in rats. *Int. J. Mol. Sci.* 16 11873–11891. 10.3390/ijms160611873 26016499 PMC4490420

[B21] Chen R. W. Qin Z. H. Ren M. Kanai H. Chalecka-Franaszek E. Leeds P. (2003). Regulation of c-Jun N-terminal kinase, p38 kinase and AP-1 DNA binding in cultured brain neurons: roles in glutamate excitotoxicity and lithium neuroprotection. *J. Neurochem.* 84 566–575. 10.1046/j.1471-4159.2003.01548.x 12558976

[B22] Chen S. N. Sun M. Zhao X. H. Yang Z. F. Liu W. X. Cao J. Y. (2019). Neuroprotection of hydroxysafflor yellow A in experimental cerebral ischemia/reperfusion injury via metabolic inhibition of phenylalanine and mitochondrial biogenesis. *Mol. Med. Rep.* 19 3009–3020. 10.3892/mmr.2019.9959 30816517 PMC6423596

[B23] Chen Y. Brandizzi F. (2013). IRE1: ER stress sensor and cell fate executor. *Trends Cell Biol.* 23 547–555. 10.1016/j.tcb.2013.06.005 23880584 PMC3818365

[B24] Chi L. Jiao D. Nan G. Yuan H. Shen J. Gao Y. (2019). miR-9-5p attenuates ischemic stroke through targeting ERMP1-mediated endoplasmic reticulum stress. *Acta Histochem.* 121:151438. 10.1016/j.acthis.2019.08.005 31500865

[B25] Clapham D. E. (2007). Calcium signaling. *Cell* 131 1047–1058. 10.1016/j.cell.2007.11.028 18083096

[B26] Cobley J. N. Fiorello M. L. Bailey D. M. (2018). 13 reasons why the brain is susceptible to oxidative stress. *Redox Biol.* 15 490–503. 10.1016/j.redox.2018.01.008 29413961 PMC5881419

[B27] Csordas G. Weaver D. Hajnoczky G. (2018). Endoplasmic reticulum-mitochondrial contactology: structure and signaling functions. *Trends Cell Biol.* 28 523–540. 10.1016/j.tcb.2018.02.009 29588129 PMC6005738

[B28] Cullinan S. Diehl J. (2004). PERK-dependent activation of Nrf2 contributes to redox homeostasis and cell survival following endoplasmic reticulum stress. *J. Biol. Chem.* 279 20108–20117. 10.1074/jbc.m314219200 14978030

[B29] Cullinan S. B. Zhang D. Hannink M. Arvisais E. Kaufman R. J. Diehl J. A. (2003). Nrf2 is a direct PERK substrate and effector of PERK-dependent cell survival. *Mol. Cell. Biol.* 23 7198–7209. 10.1128/mcb.23.20.7198-7209.2003 14517290 PMC230321

[B30] Cursio R. Colosetti P. Gugenheim J. (2015). Autophagy and liver ischemia-reperfusion injury. *Biomed Res. Int.* 2015:417590. 10.1155/2015/417590 25861623 PMC4377441

[B31] Dai M. X. Zheng X. H. Yu J. Yin T. Ma M. J. Zhang L. (2014). The impact of intermittent and repetitive cold stress exposure on endoplasmic reticulum stress and instability of atherosclerotic plaques. *Cell. Physiol. Biochem.* 34 393–404. 10.1159/000363008 25059288

[B32] Dasuri K. Zhang L. Keller J. N. (2013). Oxidative stress, neurodegeneration, and the balance of protein degradation and protein synthesis. *Free Radic. Biol. Med.* 62 170–185. 10.1016/j.freeradbiomed.2012.09.016 23000246

[B33] Datta A. Sarmah D. Mounica L. Kaur H. Kesharwani R. Verma G. (2020). Cell death pathways in ischemic stroke and targeted pharmacotherapy. *Transl. Stroke Res.* 11 1185–1202. 10.1007/s12975-020-00806-z 32219729

[B34] Datta D. Khatri P. Singh A. Saha D. R. Verma G. Raman R. (2018). *Mycobacterium fortuitum*-induced ER-MItochondrial calcium dynamics promotes calpain/caspase-12/caspase-9 mediated apoptosis in fish macrophages. *Cell Death Discov.* 4:30. 10.1038/s41420-018-0034-9 29531827 PMC5841318

[B35] de la Cadena S. G. Hernandez-Fonseca K. Camacho-Arroyo I. Massieu L. (2014). Glucose deprivation induces reticulum stress by the PERK pathway and caspase-7- and calpain-mediated caspase-12 activation. *Apoptosis* 19 414–427. 10.1007/s10495-013-0930-7 24185830

[B36] Deng J. Lu P. D. Zhang Y. Scheuner D. Kaufman R. J. Sonenberg N. (2004). Translational repression mediates activation of nuclear factor kappa B by phosphorylated translation initiation factor 2. *Mol. Cell. Biol.* 24 10161–10168. 10.1128/mcb.24.23.10161-10168.2004 15542827 PMC529034

[B37] Ding L. Ba X. H. (2015). Role of ornithine decarboxylase/polyamine pathway in focal cerebral ischemia-reperfusion injury and its mechanism in rats. *Int. J. Clin. Exp. Med.* 8 20624–20630. 26884982 PMC4723827

[B38] Ding Y. Chen M. C. Wang M. Wang M. M. Zhang T. J. Park J. S. (2014). Neuroprotection by acetyl-11-keto-beta-Boswellic acid, in ischemic brain injury involves the Nrf2/HO-1 defense pathway. *Sci. Rep.* 4:7002. 10.1038/srep07002 25384416 PMC4227012

[B39] Duong T. T. Chami B. McMahon A. C. Fong G. M. Dennis J. M. Freedman S. B. (2014). Pre-treatment with the synthetic antioxidant T-butyl bisphenol protects cerebral tissues from experimental ischemia reperfusion injury. *J. Neurochem.* 130 733–747. 10.1111/jnc.12747 24766199

[B40] Feldman H. C. Tong M. Wang L. K. Meza-Acevedo R. Gobillot T. A. Lebedev I. (2016). Structural and functional analysis of the allosteric inhibition of IRE1alpha with ATP-competitive ligands. *ACS Chem. Biol.* 11 2195–2205. 10.1021/acschembio.5b00940 27227314 PMC4992410

[B41] Feng S. Q. Zong S. Y. Liu J. X. Chen Y. Xu R. Yin X. (2019). VEGF antagonism attenuates cerebral ischemia/reperfusion-induced injury via inhibiting endoplasmic reticulum stress-mediated apoptosis. *Biol. Pharm. Bull.* 42 692–702. 10.1248/bpb.b18-00628 30828041

[B42] Gao B. Zhang X. Y. Han R. Zhang T. T. Chen C. Qin Z. H. (2013). The endoplasmic reticulum stress inhibitor salubrinal inhibits the activation of autophagy and neuroprotection induced by brain ischemic preconditioning. *Acta Pharmacol. Sin.* 34 657–666. 10.1038/aps.2013.34 23603983 PMC4002880

[B43] García de la Cadena S. Massieu L. (2016). Caspases and their role in inflammation and ischemic neuronal death. Focus on caspase-12. *Apoptosis* 21 763–777. 10.1007/s10495-016-1247-0 27142195

[B44] Gharibani P. Modi J. Menzie J. Alexandrescu A. Ma Z. Tao R. (2015). Comparison between single and combined post-treatment with S-Methyl-N,N-diethylthiolcarbamate sulfoxide and taurine following transient focal cerebral ischemia in rat brain. *Neuroscience* 300 460–473. 10.1016/j.neuroscience.2015.05.042 26022360

[B45] Gharibani P. M. Modi J. Pan C. L. Menzie J. Ma Z. Y. Chen P. C. (2013). The mechanism of taurine protection against endoplasmic reticulum stress in an animal stroke model of cerebral artery occlusion and stroke-related conditions in primary neuronal cell culture. *Adv. Exp. Med. Biol.* 776 241–258. 10.1007/978-1-4614-6093-0_23 23392887

[B46] Ghosh R. Wang L. K. Wang E. S. Perera B. G. Igbaria A. Morita S. H. (2014). Allosteric inhibition of the IRE1alpha RNase preserves cell viability and function during endoplasmic reticulum stress. *Cell* 158 534–548. 10.1016/j.cell.2014.07.002 25018104 PMC4244221

[B47] Giacomello M. Pyakurel A. Glytsou C. Scorrano L. (2020). The cell biology of mitochondrial membrane dynamics. *Nat. Rev. Mol. Cell Biol.* 21 204–224. 10.1038/s41580-020-0210-7 32071438

[B48] Gillies L. A. Kuwana T. (2014). Apoptosis regulation at the mitochondrial outer membrane. *J. Cell. Biochem.* 115 632–640. 10.1002/jcb.24709 24453042

[B49] Giorgi C. De Stefani S. D. Bononi A. Rizzuto R. Pinton P. (2009). Structural and functional link between the mitochondrial network and the endoplasmic reticulum. *Int. J. Biochem. Cell Biol.* 41 1817–1827. 10.1016/j.biocel.2009.04.010 19389485 PMC2731816

[B50] Giorgi C. Marchi S. Simoes I. C. M. Ren Z. Morciano G. Perrone M. (2018). Mitochondria and reactive oxygen species in aging and age-related diseases. *Int. Rev. Cell Mol. Biol.* 340 209–344. 10.1016/bs.ircmb.2018.05.006 30072092 PMC8127332

[B51] Glancy B. Balaban R. S. (2012). Role of mitochondrial Ca^2+^ in the regulation of cellular energetics. *Biochemistry* 51 2959–2973. 10.1021/bi2018909 22443365 PMC3332087

[B52] Go Y. M. Park H. Koval M. Orr M. Reed M. Liang Y. (2010). A key role for mitochondria in endothelial signaling by plasma cysteine/cystine redox potential. *Free Radic. Biol. Med.* 48 275–283. 10.1016/j.freeradbiomed.2009.10.050 19879942 PMC3057402

[B53] Golwala N. H. Hodenette C. Murthy S. N. Nossaman B. D. Kadowitz P. J. (2009). Vascular responses to nitrite are mediated by xanthine oxidoreductase and mitochondrial aldehyde dehydrogenase in the rat. *Can. J. Physiol. Pharmacol.* 87 1095–1101. 10.1139/y09-101 20029546

[B54] Granger D. N. Kvietys P. R. (2015). Reperfusion injury and reactive oxygen species: the evolution of a concept. *Redox Biol.* 6 524–551. 10.1016/j.redox.2015.08.020 26484802 PMC4625011

[B55] Gu Y. Ren K. W. Wang L. M. Jiang C. Z. Yao Q. Q. (2020). Rg1 in combination with mannitol protects neurons against glutamate-induced ER stress via the PERK-eIF2 alpha-ATF4 signaling pathway. *Life Sci.* 263:118559. 10.1016/j.lfs.2020.118559 33038374

[B56] Guan B. J. Krokowski D. Majumder M. Schmotzer C. L. Kimball S. R. Merrick W. C. (2014). Translational control during endoplasmic reticulum stress beyond phosphorylation of the translation initiation factor eIF2alpha. *J. Biol. Chem.* 289 12593–12611. 10.1074/jbc.m113.543215 24648524 PMC4007450

[B57] Guo M. Wang X. Zhao Y. X. Yang Q. Ding H. Y. Dong Q. (2018). Ketogenic diet improves brain ischemic tolerance and inhibits NLRP3 inflammasome activation by preventing Drp1-mediated mitochondrial fission and endoplasmic reticulum stress. *Front. Mol. Neurosci.* 11:86. 10.3389/fnmol.2018.00086 29662437 PMC5890101

[B58] Guo M. M. Qu S. B. Lu H. L. Wang W. B. He M. L. Su J. L. (2021). Biochanin A alleviates cerebral ischemia/reperfusion injury by suppressing endoplasmic reticulum stress-induced apoptosis and p38MAPK signaling pathway in vivo and in vitro. *Front. Endocrinol.* 12:646720. 10.3389/fendo.2021.646720 34322090 PMC8312488

[B59] Gupta M. K. Tahrir F. G. Knezevic T. White M. K. Gordon J. Cheung J. Y. (2016). GRP78 interacting partner Bag5 responds to ER stress and protects cardiomyocytes from ER stress-induced apoptosis. *J. Cell. Biochem.* 117 1813–1821. 10.1002/jcb.25481 26729625 PMC4909508

[B60] Han J. Back S. H. Hur J. Lin Y. H. Gildersleeve R. Shan J. X. (2013). ER-stress-induced transcriptional regulation increases protein synthesis leading to cell death. *Nat. Cell Biol.* 15 481–490. 10.1038/ncb2738 23624402 PMC3692270

[B61] Harding H. P. Zhang Y. Ron D. (1999). Protein translation and folding are coupled by an endoplasmic-reticulum-resident kinase. *Nature* 397 271–274. 10.1038/16729 9930704

[B62] Harding H. P. Zhang Y. Bertolotti A. Zeng H. Ron D. (2000). Perk is essential for translational regulation and cell survival during the unfolded protein response. *Mol. Cell* 5 897–904. 10.1016/s1097-2765(00)80330-5 10882126

[B63] Hariri H. Rogers S. Ugrankar R. Liu Y. L. Feathers J. R. Henne W. M. (2018). Lipid droplet biogenesis is spatially coordinated at ER-vacuole contacts under nutritional stress. *EMBO Rep.* 19 57–72. 10.15252/embr.201744815 29146766 PMC5757283

[B64] Hayashi T. Rizzuto R. Hajnoczky G. Su T. P. (2009). MAM: more than just a housekeeper. *Trends Cell Biol.* 19 81–88. 10.1016/j.tcb.2008.12.002 19144519 PMC2750097

[B65] Haze K. Yoshida H. Yanagi H. Yura T. Mori K. (1999). Mammalian transcription factor ATF6 is synthesized as a transmembrane protein and activated by proteolysis in response to endoplasmic reticulum stress. *Mol. Biol. Cell* 10 3787–3799. 10.1091/mbc.10.11.3787 10564271 PMC25679

[B66] Henne M. Goodman J. M. Hariri H. (2020). Spatial compartmentalization of lipid droplet biogenesis. *Biochim. Biophys. Acta Mol. Cell Biol. Lipids* 1865:158499. 10.1016/j.bbalip.2019.07.008 31352131 PMC7050823

[B67] Herker E. Vieyres G. Beller M. Krahmer N. Bohnert M. (2021). Lipid droplet contact sites in health and disease. *Trends Cell Biol.* 31 345–358. 10.1016/j.tcb.2021.01.004 33546922

[B68] Hetz C. Chevet E. Oakes S. A. (2015). Proteostasis control by the unfolded protein response. *Nat. Cell Biol.* 17 829–838. 10.1038/ncb3184 26123108 PMC5546321

[B69] Hetz C. Papa F. R. (2018). The unfolded protein response and cell fate control. *Mol. Cell* 69 169–181. 10.1016/j.molcel.2017.06.017 29107536

[B70] Hetz C. Saxena S. (2017). ER stress and the unfolded protein response in neurodegeneration. *Nat. Rev. Neurol.* 13 477–491. 10.1038/nrneurol.2017.99 28731040

[B71] Hong F. Liu B. Wu B. X. Morreall J. Roth B. Davieset C. (2017). CNPY2 is a key initiator of the PERK-CHOP pathway of the unfolded protein response. *Nat. Struct. Mol. Biol.* 24 834–839. 10.1038/nsmb.3458 28869608 PMC6102046

[B72] Hosoi T. Ogawa K. Ozawa K. (2010). Homocysteine induces X-box-binding protein 1 splicing in the mice brain. *Neurochem. Int.* 56 216–220. 10.1016/j.neuint.2009.12.005 20018221

[B73] Hoyer-Hansen M. Jaattela M. (2007). Connecting endoplasmic reticulum stress to autophagy by unfolded protein response and calcium. *Cell Death Differ.* 14 1576–1582. 10.1038/sj.cdd.4402200 17612585

[B74] Hu H. Tian M. X. Ding C. Yu S. Q. (2018). The C/EBP homologous protein (CHOP) transcription factor functions in endoplasmic reticulum stress-induced apoptosis and microbial infection. *Front. Immunol.* 9:3083. 10.3389/fimmu.2018.03083 30662442 PMC6328441

[B75] Hu L. Chen W. Tian F. Yuan C. Wang H. Yueet H. (2018). Neuroprotective role of fucoxanthin against cerebral ischemic/reperfusion injury through activation of Nrf2/HO-1 signaling. *Biomed. Pharmacother.* 106 1484–1489. 10.1016/j.biopha.2018.07.088 30119223

[B76] Huang R. R. Zhang Y. Han B. Bai Y. Zhou R. B. Gan G. M. (2017). Circular RNA HIPK2 regulates astrocyte activation via cooperation of autophagy and ER stress by targeting MIR124-2HG. *Autophagy* 13 1722–1741. 10.1080/15548627.2017.1356975 28786753 PMC5640207

[B77] Humeau J. Bravo-San Pedro J. M. Vitale I. Nuñez L. Villalobos C. Kroemer G. (2018). Calcium signaling and cell cycle: progression or death. *Cell Calcium* 70 3–15. 10.1016/j.ceca.2017.07.006 28801101

[B78] Ibrahim I. M. Abdelmalek D. H. Elfiky A. A. (2019). GRP78: a cell’s response to stress. *Life Sci.* 226 156–163. 10.1016/j.lfs.2019.04.022 30978349 PMC7094232

[B79] Jia S. Z. Xu X. W. Zhang Z. H. Chen C. Chen Y. B. Huanget S. L. (2021). Selenoprotein K deficiency-induced apoptosis: a role for calpain and the ERS pathway. *Redox Biol.* 47:102154. 10.1016/j.redox.2021.102154 34601426 PMC8495175

[B80] Jia Y. Yi L. Li Q. Q. Liu T. J. Yang S. S. (2021). LncRNA MALAT1 aggravates oxygen-glucose deprivation/reoxygenation-induced neuronal endoplasmic reticulum stress and apoptosis via the miR-195a-5p/HMGA1 axis. *Biol. Res.* 54:8. 10.1186/s40659-021-00331-9 33750458 PMC7941907

[B81] Ji Y. Q. Teng L. Zhang R. Sun J. P. Guo Y. L. (2017). NRG-1beta exerts neuroprotective effects against ischemia reperfusion-induced injury in rats through the JNK signaling pathway. *Neuroscience* 362 13–24. 10.1016/j.neuroscience.2017.08.032 28843994

[B82] Jin Z. Q. Liang J. Li J. Q. Kolattukudy P. E. (2019). Absence of MCP-induced protein 1 enhances blood-brain barrier breakdown after experimental stroke in mice. *Int. J. Mol. Sci.* 20:3214. 10.3390/ijms20133214 31261992 PMC6651107

[B83] Kalogeris T. Baines C. Krenz M. Korthuis R. J. (2016). Ischemia/reperfusion. *Compr. Physiol.* 7 113–170. 10.1002/cphy.c160006 28135002 PMC5648017

[B84] Kalogeris T. Baines C. P. Krenz M. Korthuis R. J. (2012). Cell biology of ischemia/reperfusion injury. *Int. Rev. Cell Mol. Biol.* 298 229–317. 10.1016/b978-0-12-394309-5.00006-7 22878108 PMC3904795

[B85] Kalogeris T. Bao Y. M. Korthuis R. J. (2014). Mitochondrial reactive oxygen species: a double edged sword in ischemia/reperfusion vs preconditioning. *Redox Biol.* 2 702–714. 10.1016/j.redox.2014.05.006 24944913 PMC4060303

[B86] Kaufman R. J. Malhotra J. D. (2014). Calcium trafficking integrates endoplasmic reticulum function with mitochondrial bioenergetics. *Biochim. Biophys. Acta* 1843 2233–2239. 10.1016/j.bbamcr.2014.03.022 24690484 PMC4285153

[B87] Kausar S. Wang F. Cui H. J. (2018). The role of mitochondria in reactive oxygen species generation and its implications for neurodegenerative diseases. *Cells* 7:274. 10.3390/cells7120274 30563029 PMC6316843

[B88] Khan M. S. Khan A. Ahmad S. Ahmad R. Rehman I. U. Ikram M. (2020). Inhibition of JNK alleviates chronic hypoperfusion-related ischemia induces oxidative stress and brain degeneration via Nrf2/HO-1 and NF-kappaB signaling. *Oxid. Med. Cell. Longev.* 2020:5291852. 10.1155/2020/5291852 32617137 PMC7315317

[B89] Kim I. Xu W. Reed J. C. (2008). Cell death and endoplasmic reticulum stress: disease relevance and therapeutic opportunities. *Nat. Rev. Drug. Discov.* 7 1013–1030. 10.1038/nrd2755 19043451

[B90] Korennykh A. V. Egea P. F. Korostelev A. A. Finer-Moore J. Zhang C. Shokat K. M. (2009). The unfolded protein response signals through high-order assembly of Ire1. *Nature* 457 687–693. 10.1038/nature07661 19079236 PMC2846394

[B91] Koval O. M. Nguyen E. K. Santhana V. Fidler T. P. Sebag S. C. Rasmussen T. P. (2019). Loss of MCU prevents mitochondrial fusion in G_1_-S phase and blocks cell cycle progression and proliferation. *Sci. Signal.* 12:eaav1439. 10.1126/scisignal.aav1439 31040260 PMC6768401

[B92] Kristian T. (2004). Metabolic stages, mitochondria and calcium in hypoxic/ischemic brain damage. *Cell. Calcium* 36 221–233. 10.1016/j.ceca.2004.02.016 15261478

[B93] Kuriakose D. Xiao Z. (2020). Pathophysiology and treatment of stroke: present status and future perspectives. *Int. J. Mol. Sci.* 21:7609. 10.3390/ijms21207609 33076218 PMC7589849

[B94] Kvietys P. R. Granger D. N. (2012). Role of reactive oxygen and nitrogen species in the vascular responses to inflammation. *Free. Radic. Biol. Med.* 52 556–592. 10.1016/j.freeradbiomed.2011.11.002 22154653 PMC3348846

[B95] Kwak J. H. Yang Z. G. Yoon B. He Y. X. Uhm S. Shin H. C. (2015). Blood-brain barrier-permeable fluorone-labeled dieckols acting as neuronal ER stress signaling inhibitors. *Biomaterials* 61 52–60. 10.1016/j.biomaterials.2015.04.045 25996411

[B96] Lee C. L. Veerbeek J. H. W. Rana T. K. van Rijn B. B. Burton G. J. Yung H. W. (2019). Role of endoplasmic reticulum stress in proinflammatory cytokine-mediated inhibition of trophoblast invasion in placenta-related complications of pregnancy. *Am. J. Pathol.* 189 467–478. 10.1016/j.ajpath.2018.10.015 30448406 PMC6360351

[B97] Lee K. P. Dey M. Neculai D. Cao C. Dever T. E. Sicheri F. (2008). Structure of the dual enzyme Ire1 reveals the basis for catalysis and regulation in nonconventional RNA splicing. *Cell* 132 89–100. 10.1016/j.cell.2007.10.057 18191223 PMC2276645

[B98] Lehotsky J. Urban P. Pavlikova M. Tatarková Z. Kaminska B. Kaplánet P. (2009). Molecular mechanisms leading to neuroprotection/ischemic tolerance: effect of preconditioning on the stress reaction of endoplasmic reticulum. *Cell. Mol. Neurobiol.* 29 917–925. 10.1007/s10571-009-9376-4 19283468 PMC11506296

[B99] Li M. T. Ke J. Deng Y. Q. Chen C. X. Huang Y. C. Bian Y. F. (2021). The protective effect of liquiritin in hypoxia/reoxygenation-induced disruption on blood brain barrier. *Front. Pharmacol.* 12:671783. 10.3389/fphar.2021.671783 34295249 PMC8290897

[B100] Lima B. Forrester M. T. Hess D. T. Stamler J. S. (2010). S-nitrosylation in cardiovascular signaling. *Circ. Res.* 106 633–646. 10.1161/circresaha.109.207381 20203313 PMC2891248

[B101] Lin M. Y. Ling J. Geng X. Q. Zhang J. Q. Du J. Chen L. J. (2019). RTN1-C is involved in high glucose-aggravated neuronal cell subjected to oxygen-glucose deprivation and reoxygenation injury via endoplasmic reticulum stress. *Brain Res. Bull.* 149 129–136. 10.1016/j.brainresbull.2019.04.010 31002913

[B102] Liou H. C. Boothby M. R. Finn P. W. Davidon R. Nabavi N. Zeleznik-Le N. J. (1990). A new member of the leucine zipper class of proteins that binds to the HLA DR alpha promoter. *Science* 247 1581–1584. 10.1126/science.2321018 2321018

[B103] Liu C. Y. Xu Z. H. Kaufman R. J. (2003). Structure and intermolecular interactions of the luminal dimerization domain of human IRE1alpha. *J. Biol. Chem.* 278 17680–17687. 10.1074/jbc.m300418200 12637535

[B104] Liu T. L. Liu M. N. Zhang T. J. Liu W. X. Xu H. Mu F. (2018c). Z-Guggulsterone attenuates astrocytes-mediated neuroinflammation after ischemia by inhibiting toll-like receptor 4 pathway. *J. Neurochem.* 147 803–815. 10.1111/jnc.14583 30168601

[B105] Liu F. Lu J. Manaenko A. Tang J. Hu Q. (2018b). Mitochondria in ischemic stroke: new insight and implications. *Aging Dis.* 9 924–937. 10.14336/ad.2017.1126 30271667 PMC6147588

[B106] Liu C. Fu Q. Mu R. Wang F. Zhou C. J. Zhang L. (2018a). Dexmedetomidine alleviates cerebral ischemia-reperfusion injury by inhibiting endoplasmic reticulum stress dependent apoptosis through the PERK-CHOP-Caspase-11 pathway. *Brain Res.* 1701 246–254. 10.1016/j.brainres.2018.09.007 30201260

[B107] Logsdon A. F. Lucke-Wold B. P. Nguyen L. Matsumoto R. R. Turner R. C. Rosen C. L. (2016). Salubrinal reduces oxidative stress, neuroinflammation and impulsive-like behavior in a rodent model of traumatic brain injury. *Brain Res.* 1643 140–151. 10.1016/j.brainres.2016.04.063 27131989 PMC5578618

[B108] Logue S. Cleary P. Saveljeva S. Samali A. (2013). New directions in ER stress-induced cell death. *Apoptosis* 18 537–546. 10.1007/s10495-013-0818-6 23430059

[B109] Lopata A. Kniss A. Lohr F. Rogov V. V. Dotsch V. (2020). Ubiquitination in the ERAD process. *Int. J. Mol. Sci.* 21:5369. 10.3390/ijms21155369 32731622 PMC7432864

[B110] Lopez-Hernandez B. Cena V. Posadas I. (2015). The endoplasmic reticulum stress and the HIF-1 signalling pathways are involved in the neuronal damage caused by chemical hypoxia. *Br. J. Pharmacol.* 172 2838–2851. 10.1111/bph.13095 25625917 PMC4439879

[B111] Lorenzano S. Rost N. S. Khan M. Li H. Lima F. O. Maas M. B. (2018). Oxidative stress biomarkers of brain damage: hyperacute plasma F2-isoprostane predicts infarct growth in stroke. *Stroke* 49 630–637. 10.1161/strokeaha.117.018440 29371434 PMC5828992

[B112] Luchetti F. Crinelli R. Cesarini E. Canonico B. Guidi L. Zerbinati C. (2017). Endothelial cells, endoplasmic reticulum stress and oxysterols. *Redox Biol.* 13 581–587. 10.1016/j.redox.2017.07.014 28783588 PMC5545768

[B113] Ma X. D. Zhang W. Xu C. Zhang S. S. Zhao J. X. Pan Q. (2020). Nucleotide-binding oligomerization domain protein 1 enhances oxygen-glucose deprivation and reperfusion injury in cortical neurons via activation of endoplasmic reticulum stress-mediated autophagy. *Exp. Mol. Pathol.* 117:104525. 10.1016/j.yexmp.2020.104525 32888957

[B114] Ma Y. J. Hendershot L. M. (2003). Delineation of a negative feedback regulatory loop that controls protein translation during endoplasmic reticulum stress. *J. Biol. Chem.* 278 34864–34873. 10.1074/jbc.m301107200 12840028

[B115] Mamady H. Storey K. B. (2008). Coping with the stress: expression of ATF4, ATF6, and downstream targets in organs of hibernating ground squirrels. *Arch. Biochem. Biophys.* 477 77–85. 10.1016/j.abb.2008.05.006 18541136

[B116] Man S. Xian Y. Holmes D. Matsouaka R. A. Saver J. Smith E. E. (2020). Association between thrombolytic door-to-needle time and 1-year mortality and readmission in patients with acute ischemic stroke. *JAMA* 323 2170–2184. 10.1001/jama.2020.5697 32484532 PMC7267850

[B117] Manzanero S. Santro T. Arumugam T. V. (2013). Neuronal oxidative stress in acute ischemic stroke: sources and contribution to cell injury. *Neurochem. Int.* 62 712–718. 10.1016/j.neuint.2012.11.009 23201332

[B118] Marchi S. Patergnani S. Pinton P. (2014). The endoplasmic reticulum-mitochondria connection: one touch, multiple functions. *Biochim. Biophys. Acta* 1837 461–469. 10.1016/j.bbabio.2013.10.015 24211533

[B119] Marciniak S. J. Yun C. Y. Oyadomari S. Novoa I. Zhang Y. H. Jungreis R. (2004). CHOP induces death by promoting protein synthesis and oxidation in the stressed endoplasmic reticulum. *Genes Dev.* 18 3066–3077. 10.1101/gad.1250704 15601821 PMC535917

[B120] Markouli M. Strepkos D. Papavassiliou A. G. Piperi C. (2020). Targeting of endoplasmic reticulum (ER) stress in gliomas. *Pharmacol. Res.* 157:104823. 10.1016/j.phrs.2020.104823 32305494

[B121] Martinez J. A. Zhang Z. Q. Svetlov S. I. Hayes R. L. Wang K. K. Larner S. F. (2010). Calpain and caspase processing of caspase-12 contribute to the ER stress-induced cell death pathway in differentiated PC12 cells. *Apoptosis* 15 1480–1493. 10.1007/s10495-010-0526-4 20640600

[B122] Martin-Jimenez C. A. Garcia-Vega A. Cabezas R. Aliev G. Echeverria V. González J. (2017). Astrocytes and endoplasmic reticulum stress: a bridge between obesity and neurodegenerative diseases. *Prog. Neurobiol.* 158 45–68. 10.1016/j.pneurobio.2017.08.001 28802884

[B123] Martino M. B. Jones L. Brighton B. Ehre C. Abdulah L. Davis C. W. (2013). The ER stress transducer IRE1beta is required for airway epithelial mucin production. *Mucosal Immunol.* 6 639–654. 10.1038/mi.2012.105 23168839 PMC4031691

[B124] Martire A. Lambertucci C. Pepponi R. Ferrante A. Benati N. Buccioni M. (2019). Neuroprotective potential of adenosine A1 receptor partial agonists in experimental models of cerebral ischemia. *J. Neurochem.* 149 211–230. 10.1111/jnc.14660 30614535

[B125] Mastantuono T. Battiloro L. Sabatino L. Chiurazzi M. Maro M. D. Muscariello E. (2015). Effects of citrus flavonoids against microvascular damage induced by hypoperfusion and reperfusion in rat pial circulation. *Microcirculation* 22 378–390. 10.1111/micc.12207 25944567

[B126] Matsuzaki S. Hiratsuka T. Kuwahara R. Katayama T. Tohyama M. (2010). Caspase-4 is partially cleaved by calpain via the impairment of Ca^2+^ homeostasis under the ER stress. *Neurochem. Int.* 56 352–356. 10.1016/j.neuint.2009.11.007 19931333

[B127] McCullough K. D. Martindale J. L. Klotz L. O. Aw T. Y. Holbrook N. J. (2001). Gadd153 sensitizes cells to endoplasmic reticulum stress by down-regulating Bcl2 and perturbing the cellular redox state. *Mol. Cell. Biol.* 21 1249–1259. 10.1128/mcb.21.4.1249-1259.2001 11158311 PMC99578

[B128] McQuiston A. Diehl J. A. (2017). Recent insights into PERK-dependent signaling from the stressed endoplasmic reticulum. *F1000Research* 6:1897. 10.12688/f1000research.12138.1 PMC5664976 29152224

[B129] Mei Y. Thompson M. D. Cohen R. A. Tong X. Y. (2013). Endoplasmic reticulum stress and related pathological processes. *J. Pharmacol. Biomed. Anal.* 1:1000107. 24611136 PMC3942890

[B130] Mo Z. T. Liao Y. L. Zheng J. Li W. N. (2020). Icariin protects neurons from endoplasmic reticulum stress-induced apoptosis after OGD/R injury via suppressing IRE1alpha-XBP1 signaling pathway. *Life Sci.* 255:117847. 10.1016/j.lfs.2020.117847 32470450

[B131] Mohammad-Gharibani P. Modi J. Menzie J. Genova R. Ma Z. Y. Tao R. (2014). Mode of action of S-methyl-N, N-diethylthiocarbamate sulfoxide (DETC-MeSO) as a novel therapy for stroke in a rat model. *Mol. Neurobiol.* 50 655–672. 10.1007/s12035-014-8658-0 24573692

[B132] Mohammed T. S. Tsai S. T. Hung H. Y. Hu W. F. Pang C. Y. Chen S. Y. (2020). A role for endoplasmic reticulum stress in intracerebral hemorrhage. *Cells* 9:750. 10.3390/cells9030750 32204394 PMC7140640

[B133] Morihara R. Yamashita T. Liu X. Nakano Y. Fukui Y. Sato K. (2018). Protective effect of a novel sigma-1 receptor agonist is associated with reduced endoplasmic reticulum stress in stroke male mice. *J. Neurosci. Res.* 96 1707–1716. 10.1002/jnr.24270 30102416

[B134] Morishima N. Nakanishi K. Takenouchi H. Shibata T. Yasuhiko Y. (2002). An endoplasmic reticulum stress-specific caspase cascade in apoptosis. Cytochrome c-independent activation of caspase-9 by caspase-12. *J. Biol. Chem.* 277 34287–34294. 10.1074/jbc.m204973200 12097332

[B135] Murphy M. P. (2009). How mitochondria produce reactive oxygen species. *Biochem. J.* 417 1–13. 10.1042/bj20081386 19061483 PMC2605959

[B136] Nakagawa T. Zhu H. Morishima N. Li E. Xu J. Yankner B. A. (2000). Caspase-12 mediates endoplasmic-reticulum-specific apoptosis and cytotoxicity by amyloid-beta. *Nature* 403 98–103. 10.1038/47513 10638761

[B137] Nakka V. P. Gusain A. Raghubir R. (2010). Endoplasmic reticulum stress plays critical role in brain damage after cerebral ischemia/reperfusion in rats. *Neurotox. Res.* 17 189–202. 10.1007/s12640-009-9110-5 19763736

[B138] Nakka V. P. Prakash-Babu P. Vemuganti R. (2016). Crosstalk between endoplasmic reticulum stress, oxidative stress, and autophagy: potential therapeutic targets for acute CNS injuries. *Mol. Neurobiol.* 53 532–544. 10.1007/s12035-014-9029-6 25482050 PMC4461562

[B139] Nan D. Jin H. Q. Deng J. W. Yu W. W. Liu R. Sun W. P. (2019). Cilostazol ameliorates ischemia/reperfusion-induced tight junction disruption in brain endothelial cells by inhibiting endoplasmic reticulum stress. *FASEB. J.* 33 10152–10164. 10.1096/fj.201900326r 31184927

[B140] Nishitoh H. Matsuzawa A. Tobiume K. Saegusa K. Takeda K. Inoue K. (2002). ASK1 is essential for endoplasmic reticulum stress-induced neuronal cell death triggered by expanded polyglutamine repeats. *Genes Dev.* 16 1345–1355. 10.1101/gad.992302 12050113 PMC186318

[B141] Oakes S. A. Papa F. R. (2015). The role of endoplasmic reticulum stress in human pathology. *Annu. Rev. Pathol.* 10 173–194. 10.1146/annurev-pathol-012513-104649 25387057 PMC5568783

[B142] Oh Y. S. Jun H. S. (2017). Effects of glucagon-like peptide-1 on oxidative stress and Nrf2 signaling. *Int. J. Mol. Sci.* 19:26. 10.3390/ijms19010026 29271910 PMC5795977

[B143] Ohoka N. Yoshii S. Hattori T. Onozaki K. Hayashiet H. (2005). TRB3, a novel ER stress-inducible gene, is induced via ATF4-CHOP pathway and is involved in cell death. *EMBO J.* 24 1243–1255. 10.1038/sj.emboj.7600596 15775988 PMC556400

[B144] Osada N. Kosuge Y. Ishige K. Ito Y. (2010). Characterization of neuronal and astroglial responses to ER stress in the hippocampal CA1 area in mice following transient forebrain ischemia. *Neurochem. Int.* 57 1–7. 10.1016/j.neuint.2010.03.017 20362024

[B145] Owen C. R. Kumar R. Zhang P. C. McGrath B. C. Cavener D. R. Krauseet G. S. (2005). PERK is responsible for the increased phosphorylation of eIF2alpha and the severe inhibition of protein synthesis after transient global brain ischemia. *J. Neurochem.* 94 1235–1242. 10.1111/j.1471-4159.2005.03276.x 16000157

[B146] Pahl H. L. (1999). Signal transduction from the endoplasmic reticulum to the cell nucleus. *Physiol. Rev.* 79 683–701. 10.1152/physrev.1999.79.3.683 10390516

[B147] Palam L. R. Baird T. D. Wek R. C. (2011). Phosphorylation of eIF2 facilitates ribosomal bypass of an inhibitory upstream ORF to enhance CHOP translation. *J. Biol. Chem.* 286 10939–10949. 10.1074/jbc.m110.216093 21285359 PMC3064149

[B148] Paschen W. Mengesdorf T. (2005). Endoplasmic reticulum stress response and neurodegeneration. *Cell Calcium* 38 409–415. 10.1016/j.ceca.2005.06.019 16087231

[B149] Patwardhan G. Beverly L. Siskind L. (2016). Sphingolipids and mitochondrial apoptosis. *J. Bioenerg. Biomembr.* 48 153–168. 10.1007/s10863-015-9602-3 25620271 PMC5434644

[B150] Poone G. K. Hasseldam H. Munkholm N. Rasmussen R. S. Grønberg N. V. Johansen F. F. (2015). The hypothermic influence on CHOP and Ero1-alpha in an endoplasmic reticulum stress model of cerebral ischemia. *Brain Sci.* 5 178–187. 10.3390/brainsci5020178 25989620 PMC4493463

[B151] Poustchi F. Amani H. Ahmadian Z. Niknezhad S. V. Mehrabi S. Santos H. A. (2021). Combination therapy of killing diseases by injectable hydrogels: from concept to medical applications. *Adv. Healthc. Mater.* 10:e2001571. 10.1002/adhm.202001571 33274841

[B152] Powers W. J. (2020). Acute ischemic stroke. *N. Engl. J. Med.* 383 252–260. 10.1056/nejmcp1917030 32668115

[B153] Puthalakath H. O’Reilly L. A. Gunn P. Lee L. Kelly P. N. Huntington N. D. (2007). ER stress triggers apoptosis by activating BH3-only protein Bim. *Cell* 129 1337–1349. 10.1016/j.cell.2007.04.027 17604722

[B154] Qie X. J. Wen D. Guo H. Y. Xu G. J. Liu S. Shen Q. C. (2017). Endoplasmic reticulum stress mediates methamphetamine-induced blood-brain barrier damage. *Front. Pharmacol.* 8:639. 10.3389/fphar.2017.00639 28959203 PMC5603670

[B155] Quiros P. M. Prado M. A. Zamboni N. D’Amico D. Williams R. W. Finleyet D. (2017). Multi-omics analysis identifies ATF4 as a key regulator of the mitochondrial stress response in mammals. *J. Cell Biol.* 216 2027–2045. 10.1083/jcb.201702058 28566324 PMC5496626

[B156] Raedschelders K. Ansley D. M. Chen D. D. (2012). The cellular and molecular origin of reactive oxygen species generation during myocardial ischemia and reperfusion. *Pharmacol. Ther.* 133 230–255. 10.1016/j.pharmthera.2011.11.004 22138603

[B157] Rajakumar S. Bhanupriya N. Ravi C. Nachiappan V. (2016). Endoplasmic reticulum stress and calcium imbalance are involved in cadmium-induced lipid aberrancy in *Saccharomyces cerevisiae*. *Cell Stress Chaperones* 21 895–906. 10.1007/s12192-016-0714-4 27344570 PMC5003806

[B158] Ramezani A. Nahad M. P. Faghihloo E. (2018). The role of Nrf2 transcription factor in viral infection. *J. Cell. Biochem.* 119 6366–6382. 10.1002/jcb.26897 29737559

[B159] Rao R. V. Ellerby H. M. Bredesen D. E. (2004). Coupling endoplasmic reticulum stress to the cell death program. *Cell Death Differ.* 11 372–380. 10.1038/sj.cdd.4401378 14765132

[B160] Rao R. V. Hermel E. Castro-Obregon S. del Rio G. Ellerby L. M. Ellerby H. M. (2001). Coupling endoplasmic reticulum stress to the cell death program. Mechanism of caspase activation. *J. Biol. Chem.* 276 33869–33874. 10.1074/jbc.m102225200 11448953

[B161] Raturi A. Simmen T. (2013). Where the endoplasmic reticulum and the mitochondrion tie the knot: the mitochondria-associated membrane (MAM). *Biochim. Biophys. Acta* 1833 213–224. 10.1016/j.bbamcr.2012.04.013 22575682

[B162] Rong C. Wei W. Yu-Hong T. (2020). Asperuloside exhibits a novel anti-leukemic activity by triggering ER stress-regulated apoptosis via targeting GRP78. *Biomed. Pharmacother.* 125:109819. 10.1016/j.biopha.2020.109819 32106370

[B163] Rozpedek W. Nowak A. Pytel D. Diehl J. A. Majsterek I. (2017). Molecular basis of human diseases and targeted therapy based on small-molecule inhibitors of ER stress-induced signaling pathways. *Curr. Mol. Med.* 17 118–132. 10.2174/1566524017666170306122643 28266275

[B164] Salehpour F. Farajdokht F. Mahmoudi J. Erfani M. Farhoudi M. Karimi P. (2019). Photobiomodulation and coenzyme Q10 treatments attenuate cognitive impairment associated with model of transient global brain ischemia in artificially aged mice. *Front. Cell. Neurosci.* 13:74. 10.3389/fncel.2019.00074 30983970 PMC6434313

[B165] Sanada S. Komuro I. Kitakaze M. (2011). Pathophysiology of myocardial reperfusion injury: preconditioning, postconditioning, and translational aspects of protective measures. *Am. J. Physiol. Heart. Circ. Physiol.* 301 H1723–H1741. 10.1152/ajpheart.00553.2011 21856909

[B166] Sanderson T. H. Gallaway M. Kumar R. (2015). Unfolding the unfolded protein response: unique insights into brain ischemia. *Int. J. Mol. Sci.* 16 7133–7142. 10.3390/ijms16047133 25830481 PMC4425008

[B167] Sano R. Reed J. C. (2013). ER stress-induced cell death mechanisms. *Biochim. Biophys. Acta* 1833 3460–3470. 10.1016/j.bbamcr.2013.06.028 23850759 PMC3834229

[B168] Santos-Galdiano M. Gonzalez-Rodriguez P. Font-Belmonte E. Ugidos I. F. Anuncibay-Soto B. Pérez-Rodríguez D. (2021). Celecoxib-dependent neuroprotection in a rat model of transient middle cerebral artery occlusion (tMCAO) involves modifications in unfolded protein response (UPR) and proteasome. *Mol. Neurobiol.* 58 1404–1417. 10.1007/s12035-020-02202-y 33184783

[B169] Scherz-Shouval R. Elazar Z. (2007). ROS, mitochondria and the regulation of autophagy. *Trends Cell Biol.* 17 422–427. 10.1016/j.tcb.2007.07.009 17804237

[B170] Schonthal A. (2013). Pharmacological targeting of endoplasmic reticulum stress signaling in cancer. *Biochem. Pharmacol.* 85 653–666. 10.1016/j.bcp.2012.09.012 23000916

[B171] Schonthal A. H. (2012). Endoplasmic reticulum stress: its role in disease and novel prospects for therapy. *Scientifica* 2012:857516. 10.6064/2012/857516 24278747 PMC3820435

[B172] Shai N. Yifrach E. van R. C. Cohen N. Bibi C. IJlst L. (2018). Systematic mapping of contact sites reveals tethers and a function for the peroxisome-mitochondria contact. *Nat. Commun.* 9:1761. 10.1038/s41467-018-03957-8 PMC5932058 29720625

[B173] Shen Y. T. Li R. Yu S. Zhao Q. Wang Z. R. Sheng H. X. (2021). Activation of the ATF6 (Activating Transcription Factor 6) signaling pathway in neurons improves outcome after cardiac arrest in mice. *J. Am. Heart Assoc.* 10:e020216. 10.1161/jaha.120.020216 34111943 PMC8477867

[B174] Shi W. Z. Tian Y. Li J. (2019). GCN2 suppression attenuates cerebral ischemia in mice by reducing apoptosis and endoplasmic reticulum (ER) stress through the blockage of FoxO3a-regulated ROS production. *Biochem. Biophys. Res. Commun.* 516 285–292. 10.1016/j.bbrc.2019.05.181 31255283

[B175] Shibata M. Hattori H. Sasaki T. Gotoh J. Hamada J. Fukuuchi Y. (2003). Activation of caspase-12 by endoplasmic reticulum stress induced by transient middle cerebral artery occlusion in mice. *Neuroscience* 118 491–499. 10.1016/s0306-4522(02)00910-7 12699784

[B176] Sprenkle N. T. Sims S. G. Sanchez C. L. Meares G. P. (2017). Endoplasmic reticulum stress and inflammation in the central nervous system. *Mol. Neurodegener.* 12:42. 10.1186/s13024-017-0183-y 28545479 PMC5445486

[B177] Starck S. R. Tsai J. C. Chen K. Shodiya M. Wang L. Yahiro K. (2016). Translation from the 5’ untranslated region shapes the integrated stress response. *Science* 351:aad3867. 10.1126/science.aad3867 26823435 PMC4882168

[B178] Stefan C. J. Manford A. G. Baird D. Yamada-Hanff J. Mao Y. Emr S. D. (2011). Osh proteins regulate phosphoinositide metabolism at ER-plasma membrane contact sites. *Cell* 144 389–401. 10.1016/j.cell.2010.12.034 21295699

[B179] Stroud D. A. Oeljeklaus S. Wiese S. Bohnert M. Lewandrowski U. Sickmann A. (2011). Composition and topology of the endoplasmic reticulum-mitochondria encounter structure. *J. Mol. Biol.* 413 743–750. 10.1016/j.jmb.2011.09.012 21945531

[B180] Su L. Zhang R. Chen Y. Zhu Z. Ma C. (2017). Raf kinase inhibitor protein attenuates ischemic-induced microglia cell apoptosis and activation through NF-kappaB pathway. *Cell. Physiol. Biochem.* 41 1125–1134. 10.1159/000464119 28245468

[B181] Sun B. Z. Chen L. Wu Q. Wang H. L. Wei X. B. Xiang Y. X. (2014). Suppression of inflammatory response by flurbiprofen following focal cerebral ischemia involves the NF-kappaB signaling pathway. *Int. J. Clin. Exp. Med.* 7 3087–3095. 25356186 PMC4211836

[B182] Sun M. S. Jin H. Sun X. Huang S. Zhang F. L. Guo Z. N. (2018). Free radical damage in ischemia-reperfusion injury: an obstacle in acute ischemic stroke after revascularization therapy. *Oxid. Med. Cell. Longev.* 2018:3804979. 10.1155/2018/3804979 29770166 PMC5892600

[B183] Szegezdi E. Logue S. E. Gorman A. M. Samali A. (2006). Mediators of endoplasmic reticulum stress-induced apoptosis. *EMBO Rep.* 7 880–885. 10.1038/sj.embor.7400779 16953201 PMC1559676

[B184] Tajiri S. Oyadomari S. Yano S. Morioka M. Gotoh T. Hamada J. I. (2004). Ischemia-induced neuronal cell death is mediated by the endoplasmic reticulum stress pathway involving CHOP. *Cell Death Differ.* 11 403–415. 10.1038/sj.cdd.4401365 14752508

[B185] Ten V. Galkin A. (2019). Mechanism of mitochondrial complex I damage in brain ischemia/reperfusion injury. A hypothesis. *Mol. Cell. Neurosci.* 100:103408. 10.1016/j.mcn.2019.103408 31494262 PMC11500760

[B186] Thuerauf D. J. Morrison L. E. Hoover H. Glembotski C. C. (2002). Coordination of ATF6-mediated transcription and ATF6 degradation by a domain that is shared with the viral transcription factor, VP16. *J. Biol. Chem.* 277 20734–20739. 10.1074/jbc.m201749200 11909875

[B187] Tian Y. Su Y. Ye Q. Chen L. Yuan F. Wang Z. Y. (2020). Silencing of TXNIP alleviated oxidative stress injury by regulating MAPK-Nrf2 axis in ischemic stroke. *Neurochem. Res.* 45 428–436. 10.1007/s11064-019-02933-y 31858374

[B188] Toulmay A. Prinz W. A. (2011). Lipid transfer and signaling at organelle contact sites: the tip of the iceberg. *Curr. Opin. Cell Biol.* 23 458–463. 10.1016/j.ceb.2011.04.006 21555211 PMC3148286

[B189] Travers K. J. Patil C. K. Wodicka L. Lockhart D. J. Weissman J. S. Walter P. (2000). Functional and genomic analyses reveal an essential coordination between the unfolded protein response and ER-associated degradation. *Cell* 101 249–258. 10.1016/s0092-8674(00)80835-1 10847680

[B190] Tripathi M. Zhang C. W. Singh B. K. Sinha R. A. Moe K. T. Desilva D. A. (2016). Hyperhomocysteinemia causes ER stress and impaired autophagy that is reversed by vitamin B supplementation. *Cell Death Dis.* 7:e2513. 10.1038/cddis.2016.374 27929536 PMC5260994

[B191] Ugrankar R. Bowerman J. Hariri H. Chandra M. Chen K. Bossanyi M. F. (2019). *Drosophila* Snazarus regulates a lipid droplet population at plasma membrane-droplet contacts in adipocytes. *Dev. Cell* 50 557–572.e5. 10.1016/j.devcel.2019.07.021 31422916 PMC7446143

[B192] Urra H. Hetz C. (2017). Fine-tuning PERK signaling to control cell fate under stress. *Nat. Struct. Mol. Biol.* 24 789–790. 10.1038/nsmb.3478 28981072

[B193] Uzdensky A. B. (2019). Apoptosis regulation in the penumbra after ischemic stroke: expression of pro- and antiapoptotic proteins. *Apoptosis* 24 687–702. 10.1007/s10495-019-01556-6 31256300

[B194] Valko M. Leibfritz D. Moncol J. Cronin M. T. Mazur M. Telser J. (2007). Free radicals and antioxidants in normal physiological functions and human disease. *Int. J. Biochem. Cell Biol.* 39 44–84. 10.1016/j.biocel.2006.07.001 16978905

[B195] Valm A. M. Cohen S. Legant W. R. Melunis J. Hershberg U. Wait E. (2017). Applying systems-level spectral imaging and analysis to reveal the organelle interactome. *Nature* 546 162–167. 10.1038/nature22369 28538724 PMC5536967

[B196] Vicente-Gutierrez C. Bonora N. Bobo-Jimenez V. Jimenez-Blasco D. Lopez-Fabuel I. Fernandez E. (2019). Astrocytic mitochondrial ROS modulate brain metabolism and mouse behaviour. *Nat. Metab.* 1 201–211. 10.1038/s42255-018-0031-6 32694785

[B197] Volmer R. van der Ploeg K. Ron D. (2013). Membrane lipid saturation activates endoplasmic reticulum unfolded protein response transducers through their transmembrane domains. *Proc. Natl. Acad. Sci. U.S.A.* 110 4628–4633. 10.1073/pnas.1217611110 23487760 PMC3606975

[B198] Waldherr S. M. Strovas T. J. Vadset T. A. Liachko N. F. Kraemer B. C. (2019). Constitutive XBP-1s-mediated activation of the endoplasmic reticulum unfolded protein response protects against pathological tau. *Nat. Commun.* 10:4443. 10.1038/s41467-019-12070-3 31570707 PMC6768869

[B199] Walter P. Ron D. (2011). The unfolded protein response: from stress pathway to homeostatic regulation. *Science* 334 1081–1086. 10.1126/science.1209038 22116877

[B200] Wang M. Hayashi H. Horinokita I. Asada M. Iwatani Y. Liu J. X. (2021). Neuroprotective effects of senkyunolide I against glutamate-induced cells death by attenuating JNK/caspase-3 activation and apoptosis. *Biomed. Pharmacother.* 140:111696. 10.1016/j.biopha.2021.111696 34044281

[B201] Wang X. H. Zhao J. Guo H. M. Fan Q. Q. (2019). CFLAR is a critical regulator of cerebral ischaemia-reperfusion injury through regulating inflammation and endoplasmic reticulum (ER) stress. *Biomed. Pharmacother.* 117:109155. 10.1016/j.biopha.2019.109155 31387178

[B202] Wang Z. F. Zhang C. Y. Hong Z. H. Chen H. X. Chen W. F. Chen G. F. (2013). C/EBP homologous protein (CHOP) mediates neuronal apoptosis in rats with spinal cord injury. *Exp. Ther. Med.* 5 107–111. 10.3892/etm.2012.745 23251250 PMC3523958

[B203] Waza A. A. Hamid Z. Bhat S. A. Naseer U. D. Bhat M. Ganai B. (2018). Relaxin protects cardiomyocytes against hypoxia-induced damage in in-vitro conditions: involvement of Nrf2/HO-1 signaling pathway. *Life Sci.* 213 25–31. 10.1016/j.lfs.2018.08.059 30176248

[B204] Wei J. L. Wu X. Q. Luo P. Yue K. Y. Yu Y. Pu J. N. (2019). Homer1a attenuates endoplasmic reticulum stress-induced mitochondrial stress after ischemic reperfusion injury by inhibiting the PERK pathway. *Front. Cell. Neurosci.* 13:101. 10.3389/fncel.2019.00101 30930751 PMC6428733

[B205] Weidinger A. Kozlov A. V. (2015). Biological activities of reactive oxygen and nitrogen species: oxidative stress versus signal transduction. *Biomolecules* 5 472–484. 10.3390/biom5020472 25884116 PMC4496681

[B206] Wong M. Y. DiChiara A. S. Suen P. H. Chen K. Doan N. D. Shoulders M. D. (2018). Adapting secretory proteostasis and function through the unfolded protein response. *Curr. Top. Microbiol. Immunol.* 414 1–25. 10.1007/82_2017_56 28929194 PMC5860992

[B207] Wu F. Qiu J. Fan Y. Zhang Q. L. Cheng B. H. Wu Y. L. (2018). Apelin-13 attenuates ER stress-mediated neuronal apoptosis by activating Galphai/Galphaq-CK2 signaling in ischemic stroke. *Exp. Neurol.* 302 136–144. 10.1016/j.expneurol.2018.01.006 29337146

[B208] Wu F. Zhang R. M. Feng Q. Z. Cheng H. J. Xue J. J. Chen J. (2020). (−)-Clausenamide alleviated ER stress and apoptosis induced by OGD/R in primary neuron cultures. *Neurol. Res.* 42 730–738. 10.1080/01616412.2020.1771040 32588767

[B209] Wu J. Rutkowski D. T. Dubois M. Swathirajan J. Saunders T. Wang J. Y. (2007). ATF6alpha optimizes long-term endoplasmic reticulum function to protect cells from chronic stress. *Dev. Cell* 13 351–364. 10.1016/j.devcel.2007.07.005 17765679

[B210] Wu M. Y. Yiang G. T. Liao W. T. Tsai A. P. Cheng Y. L. Cheng P. W. (2018). Current mechanistic concepts in ischemia and reperfusion injury. *Cell. Physiol. Biochem.* 46 1650–1667. 10.1159/000489241 29694958

[B211] Wu Y. Whiteus C. Xu C. S. Hayworth K. J. Weinberg R. J. Hess H. F. (2017b). Contacts between the endoplasmic reticulum and other membranes in neurons. *Proc. Natl. Acad. Sci. U.S.A.* 114 E4859–E4867. 10.1073/pnas.1701078114 28559323 PMC5474793

[B212] Wu Y. Wang X. Zhou X. Cheng B. Li G. Bai B. (2017a). Temporal expression of Apelin/Apelin receptor in ischemic stroke and its therapeutic potential. *Front. Mol. Neurosci.* 10:1. 10.3389/fnmol.2017.00001 28167898 PMC5253351

[B213] Xia P. P. Zhang F. Yuan Y. J. Chen C. Huang Y. Li L. Y. (2020). ALDH 2 conferred neuroprotection on cerebral ischemic injury by alleviating mitochondria-related apoptosis through JNK/caspase-3 signing pathway. *Int. J. Biol. Sci.* 16 1303–1323. 10.7150/ijbs.38962 32210721 PMC7085232

[B214] Xie P. Ren Z. K. Lv J. Hu Y. M. Guan Z. Z. Yu W. F. (2020). Berberine ameliorates oxygen-glucose deprivation/reperfusion-induced apoptosis by inhibiting endoplasmic reticulum stress and autophagy in PC12 cells. *Curr. Med. Sci.* 40 1047–1056. 10.1007/s11596-020-2286-x 33428132

[B215] Xin J. H. Ma X. X. Chen W. Y. Zhou W. Dong H. P. Wang Z. (2021). Regulation of blood-brain barrier permeability by Salvinorin A via alleviating endoplasmic reticulum stress in brain endothelial cell after ischemia stroke. *Neurochem. Int.* 149:105093. 10.1016/j.neuint.2021.105093 34097989

[B216] Xin Q. Ji B. Cheng B. Wang C. Liu H. Chen X. (2014). Endoplasmic reticulum stress in cerebral ischemia. *Neurochem. Int.* 68 18–27. 10.1016/j.neuint.2014.02.001 24560721

[B217] Xu Q. X. Zhao B. Ye Y. Z. Li Y. N. Zhang Y. G. Xiong X. X. (2021). Relevant mediators involved in and therapies targeting the inflammatory response induced by activation of the NLRP3 inflammasome in ischemic stroke. *J. Neuroinflammation* 18:123. 10.1186/s12974-021-02137-8 34059091 PMC8166383

[B218] Xu W. L. Li T. Gao L. S. Zheng J. W. Yan J. Zhang J. M. (2019). Apelin-13/APJ system attenuates early brain injury via suppression of endoplasmic reticulum stress-associated TXNIP/NLRP3 inflammasome activation and oxidative stress in a AMPK-dependent manner after subarachnoid hemorrhage in rats. *J. Neuroinflammation* 16:247. 10.1186/s12974-019-1620-3 31791369 PMC6889224

[B219] Yamaguchi H. Wang H. G. (2004). CHOP is involved in endoplasmic reticulum stress-induced apoptosis by enhancing DR5 expression in human carcinoma cells. *J. Biol. Chem.* 279 45495–45502. 10.1074/jbc.m406933200 15322075

[B220] Yamamoto K. Sato T. Matsui T. Sato M. Okada T. Yoshida H. (2007). Transcriptional induction of mammalian ER quality control proteins is mediated by single or combined action of ATF6alpha and XBP1. *Dev. Cell* 13 365–376. 10.1016/j.devcel.2007.07.018 17765680

[B221] Yang J. P. Wang Z. R. Liu X. Y. Lu P. C. (2021). Modulation of vascular integrity and neuroinflammation by peroxiredoxin 4 following cerebral ischemia-reperfusion injury. *Microvasc. Res.* 135:104144. 10.1016/j.mvr.2021.104144 33515567

[B222] Yang J. W. Hu Z. P. (2015). Neuroprotective effects of atorvastatin against cerebral ischemia/reperfusion injury through the inhibition of endoplasmic reticulum stress. *Neural. Regen. Res.* 10 1239–1244. 10.4103/1673-5374.162755 26487850 PMC4590235

[B223] Yang W. Paschen W. (2016). Unfolded protein response in brain ischemia: a timely update. *J. Cereb. Blood Flow Metab.* 36 2044–2050. 10.1177/0271678x16674488 27733676 PMC5363674

[B224] Yang Y. Liu L. Naik I. Braunstein Z. Zhong J. X. Ren B. X. (2017). Transcription factor C/EBP homologous protein in health and diseases. *Front. Immunol.* 8:1612. 10.3389/fimmu.2017.01612 29230213 PMC5712004

[B225] Yao W. J. Yang X. W. Zhu J. Y. Gao B. Shi H. T. Xu L. P. (2018). IRE1alpha siRNA relieves endoplasmic reticulum stress-induced apoptosis and alleviates diabetic peripheral neuropathy in vivo and in vitro. *Sci. Rep.* 8:2579. 10.1038/s41598-018-20950-9 29416111 PMC5803253

[B226] Ye J. Rawson R. B. Komuro R. Chen X. Davé U. P. Prywes R. (2000). ER stress induces cleavage of membrane-bound ATF6 by the same proteases that process SREBPs. *Mol. Cell* 6 1355–1364. 10.1016/s1097-2765(00)00133-7 11163209

[B227] Ye Z. Wang N. Xia P. P. Wang E. Liao J. Guo Q. L. (2013). Parecoxib suppresses CHOP and Foxo1 nuclear translocation, but increases GRP78 levels in a rat model of focal ischemia. *Neurochem. Res.* 38 686–693. 10.1007/s11064-012-0953-4 23325452

[B228] Yoneda T. Imaizumi K. Oono K. Yui D. Gomi F. Katayama T. (2001). Activation of caspase-12, an endoplastic reticulum (ER) resident caspase, through tumor necrosis factor receptor-associated factor 2-dependent mechanism in response to the ER stress. *J. Biol. Chem.* 276 13935–13940. 10.1074/jbc.m010677200 11278723

[B229] Yoshida H. Matsui T. Hosokawa N. Kaufman R. J. Nagata K. Mori K. (2003). A time-dependent phase shift in the mammalian unfolded protein response. *Dev. Cell* 4 265–271. 10.1016/s1534-5807(03)00022-4 12586069

[B230] Yoshida H. Okada T. Haze K. Yanagi H. Yura T. Negishi M. (2001b). Endoplasmic reticulum stress-induced formation of transcription factor complex ERSF including NF-Y (CBF) and activating transcription factors 6alpha and 6beta that activates the mammalian unfolded protein response. *Mol. Cell. Biol.* 21 1239–1248. 10.1128/mcb.21.4.1239-1248.2001 11158310 PMC99577

[B231] Yoshida H. Matsui T. Yamamoto A. Okada T. Mori K. (2001a). XBP1 mRNA is induced by ATF6 and spliced by IRE1 in response to ER stress to produce a highly active transcription factor. *Cell* 107 881–891. 10.1016/s0092-8674(01)00611-0 11779464

[B232] Yoshida H. Okada T. Haze K. Yanagi H. Yura T. Negishi M. (2000). ATF6 activated by proteolysis binds in the presence of NF-Y (CBF) directly to the cis-acting element responsible for the mammalian unfolded protein response. *Mol. Cell. Biol.* 20 6755–6767. 10.1128/mcb.20.18.6755-6767.2000 10958673 PMC86199

[B233] Yoshikawa A. Kamide T. Hashida K. Ta H. M. Inahata Y. Takarada-Iemata M. (2015). Deletion of Atf6alpha impairs astroglial activation and enhances neuronal death following brain ischemia in mice. *J. Neurochem.* 132 342–353. 10.1111/jnc.12981 25351847

[B234] Young S. K. Wek R. C. (2016). Upstream open reading frames differentially regulate gene-specific translation in the integrated stress response. *J. Biol. Chem.* 291 16927–16935. 10.1074/jbc.r116.733899 27358398 PMC5016099

[B235] Yu Z. Sheng H. X. Liu S. Zhao S. L. Glembotski C. C. Warneret D. S. (2017). Activation of the ATF6 branch of the unfolded protein response in neurons improves stroke outcome. *J. Cereb. Blood Flow Metab.* 37 1069–1079. 10.1177/0271678x16650218 27217380 PMC5363481

[B236] Yue S. Zhu J. J. Zhang M. Li C. Y. Zhou X. L. Zhou M. (2016). The myeloid heat shock transcription factor 1/beta-catenin axis regulates NLR family, pyrin domain-containing 3 inflammasome activation in mouse liver ischemia/reperfusion injury. *Hepatology* 64 1683–1698. 10.1002/hep.28739 27474884 PMC5074868

[B237] Zeeshan H. Lee G. H. Kim H. R. Chae H. J. (2016). Endoplasmic reticulum stress and associated ROS. *Int. J. Mol. Sci.* 17:327. 10.3390/ijms17030327 26950115 PMC4813189

[B238] Zhang K. Kaufman R. J. (2006). The unfolded protein response: a stress signaling pathway critical for health and disease. *Neurology* 66 S102–S109. 10.1212/01.wnl.0000192306.98198.ec 16432136

[B239] Zhang M. M. Zhou D. Z. Ouyang Z. Yu M. Q. Jiang Y. G. (2020). Sphingosine kinase 1 promotes cerebral ischemia-reperfusion injury through inducing ER stress and activating the NF-kappaB signaling pathway. *J. Cell. Physiol.* 235 6605–6614. 10.1002/jcp.29546 31985036

[B240] Zhang Q. Y. Wang Z. J. Sun D. M. Wang Y. Xu P. Wu W. J. (2017). Novel therapeutic effects of leonurine on ischemic stroke: new mechanisms of BBB integrity. *Oxid. Med. Cell. Longev.* 2017:7150376. 10.1155/2017/7150376 28690765 PMC5485366

[B241] Zhang Y. Zhou H. Wu W. B. Shi C. Hu S. Y. Yin T. (2016). Liraglutide protects cardiac microvascular endothelial cells against hypoxia/reoxygenation injury through the suppression of the SR-Ca^2+^-XO-ROS axis via activation of the GLP-1R/PI3K/Akt/survivin pathways. *Free Radic. Biol. Med.* 95 278–292. 10.1016/j.freeradbiomed.2016.03.035 27038735

[B242] Zhao L. Li H. M. Gao Q. Xu J. Zhu Y. J. Zhai M. L. (2021). Berberine attenuates cerebral ischemia-reperfusion injury induced neuronal apoptosis by down-regulating the CNPY2 signaling pathway. *Front. Pharmacol.* 12:609693. 10.3389/fphar.2021.609693 33995012 PMC8113774

[B243] Zhao Q. Wang X. Chen A. Cheng X. Zhang G. Sun J. (2018). Rhein protects against cerebral ischemic/reperfusioninduced oxidative stress and apoptosis in rats. *Int. J. Mol. Med.* 41 2802–2812. 10.3892/ijmm.2018.3488 29436613 PMC5846655

[B244] Zhao Y. N. Li J. M. Chen C. X. Zhang P. Li S. X. (2015). Hypertension-mediated enhancement of JNK activation in association with endoplasmic reticulum stress in rat model hippocampus with cerebral ischemia-reperfusion. *Genet. Mol. Res.* 14 10980–10990. 10.4238/2015.september.21.10 26400327

[B245] Zhou L. Ao L. Y. Yan Y. Y. Li W. T. Ye A. Q. Li C. Y. (2019). JLX001 ameliorates ischemia/reperfusion injury by reducing neuronal apoptosis via down-regulating JNK signaling pathway. *Neuroscience* 418 189–204. 10.1016/j.neuroscience.2019.08.053 31487541

[B246] Zhu C. Johansen F. E. Prywes R. (1997). Interaction of ATF6 and serum response factor. *Mol. Cell. Biol.* 17 4957–4966. 10.1128/mcb.17.9.4957 9271374 PMC232347

[B247] Zhu H. Y. Zhu H. Y. Xiao S. P. Su H. Y. Xie C. L. Ma Y. W. (2012). Activation and crosstalk between the endoplasmic reticulum road and JNK pathway in ischemia-reperfusion brain injury. *Acta. Neurochir.* 154 1197–1203. 22638597 10.1007/s00701-012-1396-z

[B248] Zhu L. Yang K. Wang X. E. Wang X. Wang C. C. (2014). A novel reaction of peroxiredoxin 4 towards substrates in oxidative protein folding. *PLoS One* 9:e105529. 10.1371/journal.pone.0105529 25137134 PMC4138195

[B249] Zhu Y. Yu J. B. Gong J. B. Shen J. Ye D. Chenget D. X. (2021). PTP1B inhibitor alleviates deleterious microglial activation and neuronal injury after ischemic stroke by modulating the ER stress-autophagy axis via PERK signaling in microglia. *Aging* 13 3405–3427. 10.18632/aging.202272 33495405 PMC7906217

[B250] Zrzavy T. Machado-Santos J. Christine S. Baumgartner C. Weiner H. L. Butovskyet O. (2018). Dominant role of microglial and macrophage innate immune responses in human ischemic infarcts. *Brain Pathol.* 28 791–805. 10.1111/bpa.12583 29222823 PMC6334527

